# Supplementary material for: Deciphering cell lineage specification of human lung adenocarcinoma with single-cell RNA sequencing
Source: Nat Commun. 2021 Nov 11;12:6500. doi: 10.1038/s41467-021-26770-2 (PMC8586023; doi:10.1038/s41467-021-26770-2)
Supplement: Supplementary file 1 — Supplementary Information [file 41467_2021_26770_MOESM1_ESM.pdf]

Deciphering cell lineage specification of human lung adenocarcinoma with single-cell RNA sequencing

Zhoufeng Wang[1,2,3,11], Zhe Li[4,11], Kun Zhou[5,6,11], Chengdi Wang[1,11], Lili Jiang[7], Li Zhang[1], Ying Yang[1], Wenxin Luo[1], Wenliang Qiao[8], Gang Wang[2], Yinyun Ni[1], Shuiping Dai[9], Tingting Guo[1], Guiyi Ji[10], Minjie Xu[4], Yiying Liu[4], Zhixi Su[4], Guowei Che[6]✉, and Weimin Li[1,2,3]✉

Affiliations:

[1] Department of Respiratory and Critical Care Medicine, Frontiers Science Center for Disease-related Molecular Network, West China Hospital, Sichuan University, Chengdu, Sichuan, China

[2] Precision Medicine Research Center, West China Hospital, Sichuan University, Chengdu, Sichuan, China

[3] Research Units of West China, Chinese Academy of Medical Sciences, West China Hospital, Chengdu, Sichuan, China

[4] Singlera Genomics Ltd., Shanghai, China

[5] Department of Thoracic Surgery, The First Affiliated Hospital, Zhejiang University School of Medicine, Hangzhou, Zhejiang, China

[6] Department of Thoracic Surgery, West China Hospital, Sichuan University, Chengdu, Sichuan, China

[7] Department of Pathology, West China Hospital of Sichuan University, Chengdu, China

[8] Lung Cancer Center, West China Hospital Sichuan University, Chengdu, Sichuan, China

[9] Center of Gerontology and Geriatrics, West China Hospital, Sichuan University, Chengdu, Sichuan, China

[10] Health Management Center, West China Hospital, Sichuan University, Chengdu, Sichuan, China

[11] These authors contributed equally: Zhoufeng Wang, Zhe Li, Kun Zhou, Chengdi Wang

✉ Correspondence to: cheguowei_hx@aliyun.com (G. C.), Phone: +86-189-8060-1890; weimi003@scu.edu.cn (W. L.), Phone: +86-189-8060-1009, Fax: 028-85164165

This PDF file includes:

Figs. S1 to S23

Table S1

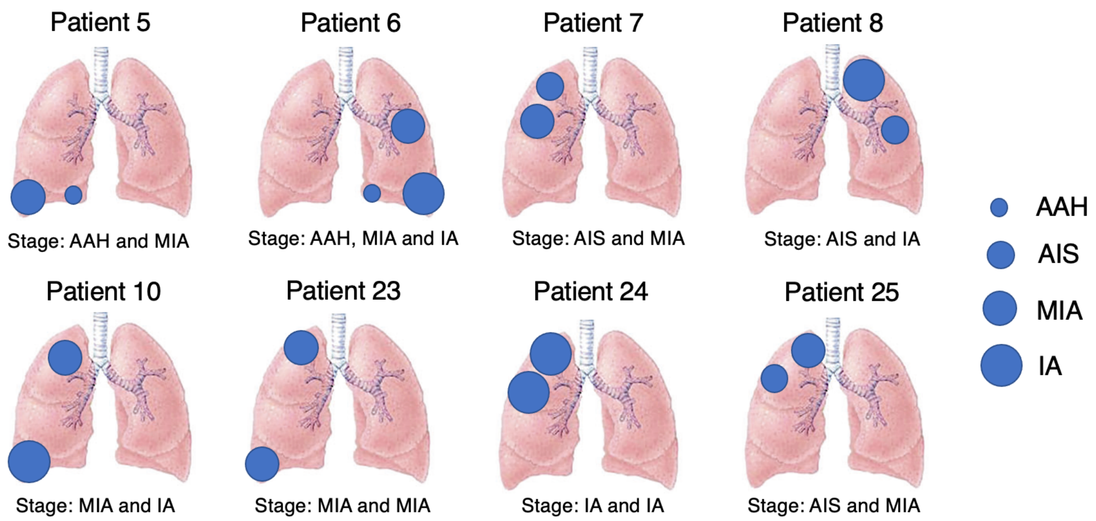

Fig. S1. Characteristics of eight lung cancer patients with multiple nodules included in this study. Patients 5 to 10 were in the discovery group, while patients 23 to 25 were in the validation group. AAH: atypical adenomatous hyperplasia; AIS: adenocarcinoma in situ; MIA: minimally invasive adenocarcinoma; IA: invasive adenocarcinoma

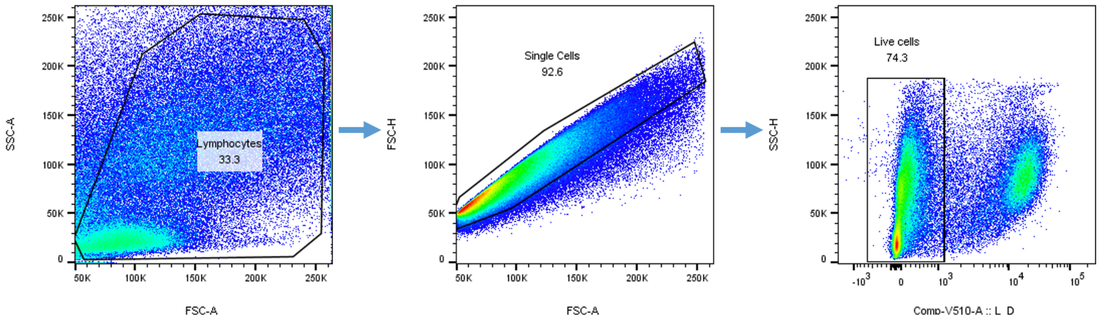

Fig. S2. A representative flow cytometric plot of gated live cells from normal lung tissues and tumor tissues, sorted for scRNA-seq library construction.

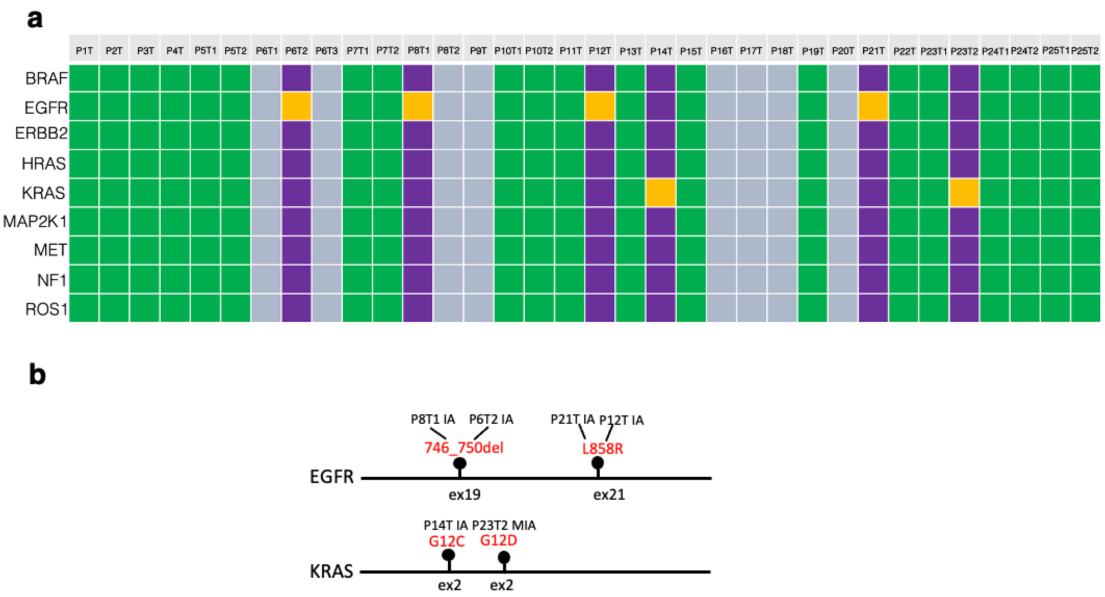

Fig. S3. Somatic alterations in different histologic stages of LUAD patients. (a) Driver gene mutations in 26 tissues from 18 LUAD patients. The labels above indicate tumor identifiers. Yellow cells represent mutated genes. Green color cells mean wild type of the given patients. Purple color cells mean that driver gene mutations were detected in the given patients. Grey color cells mean no specimens available. (b) Schematics of known driver oncogenes showing somatic variants observed in this dataset. Each lollipop indicates a different somatic variant.

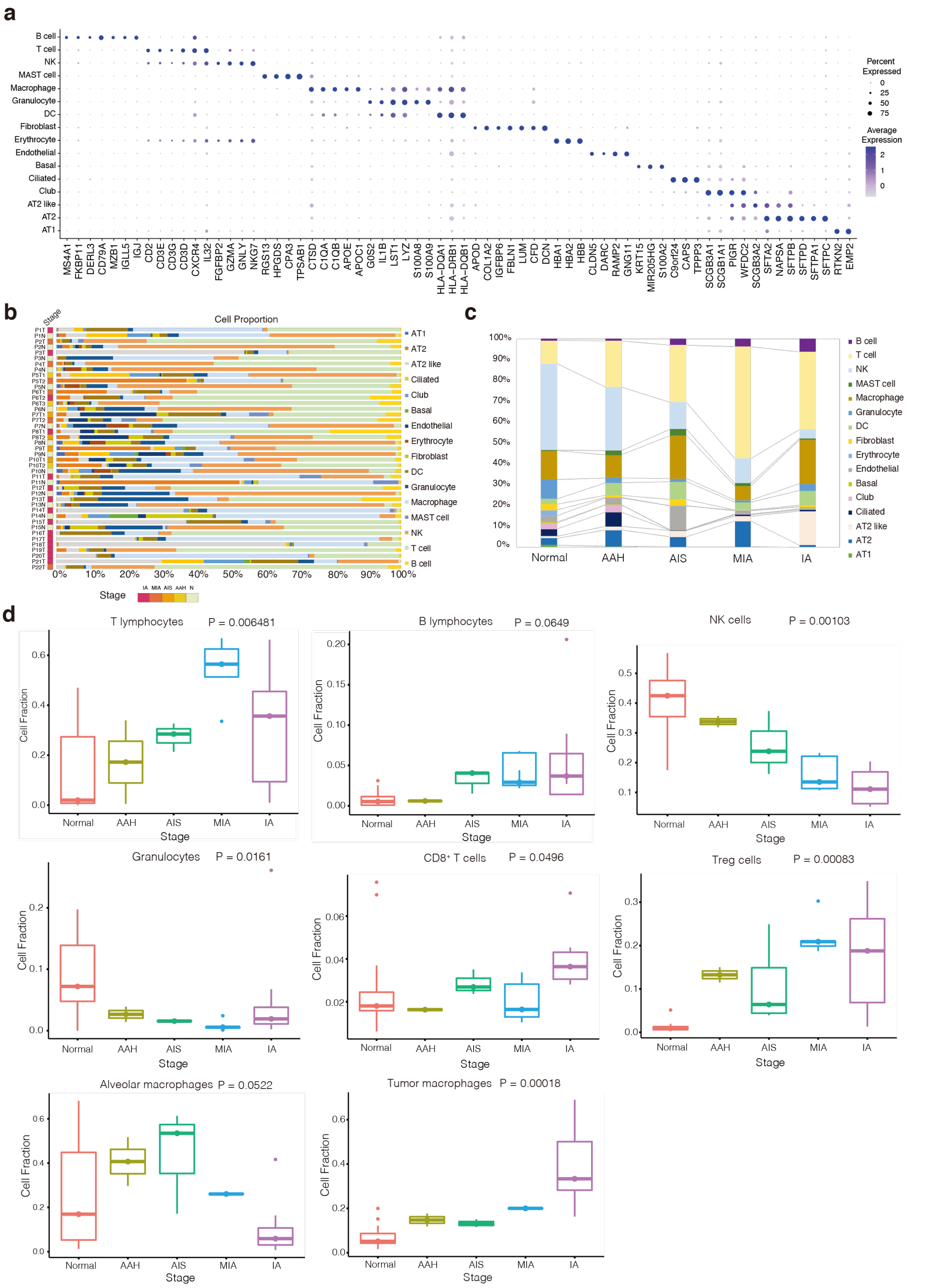

Fig. S4. Expression of marker genes for the cell types and characterization of cell proportions in normal lung and LUAD tissues from different histologic stages. (a) Dot plot showing the marker genes of each cell type cluster defined in Fig. 1b. (b) Proportions of cells in each patient that belonged to the 16 major cell types identified in the study. (c) Percentages of cell proportions in each cell type from normal lung tissues and tissues in different histologic stages. (d) Proportion of immune cell types varied among normal lung tissues (n=14 replicates) and tissues from AAH (n=2 replicates), AIS (n=3 replicates), MIA (n=5 replicates) to IA (n=13 replicates). Differences were assessed for significance using the Kruskal-Wallis rank test, one-sided test. Center line: Median; Box limits: Upper and lower quartiles. Source data are provided as a Source Data file.

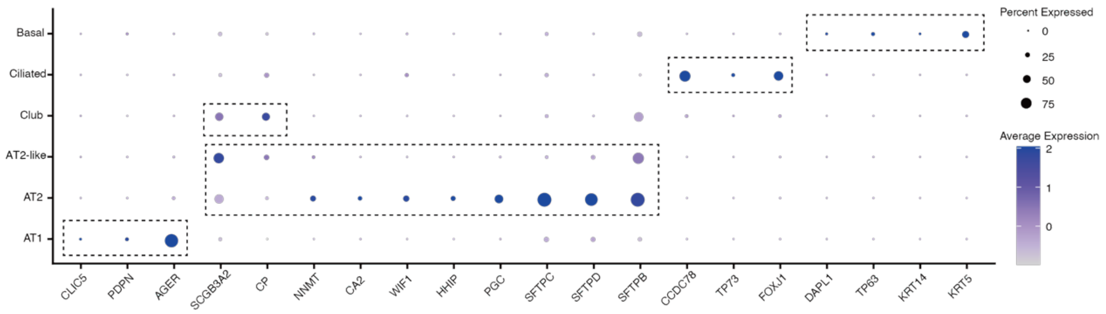

Fig. S5. Dot plot of differentially expressed epithelial features labeled based on cell clusters.

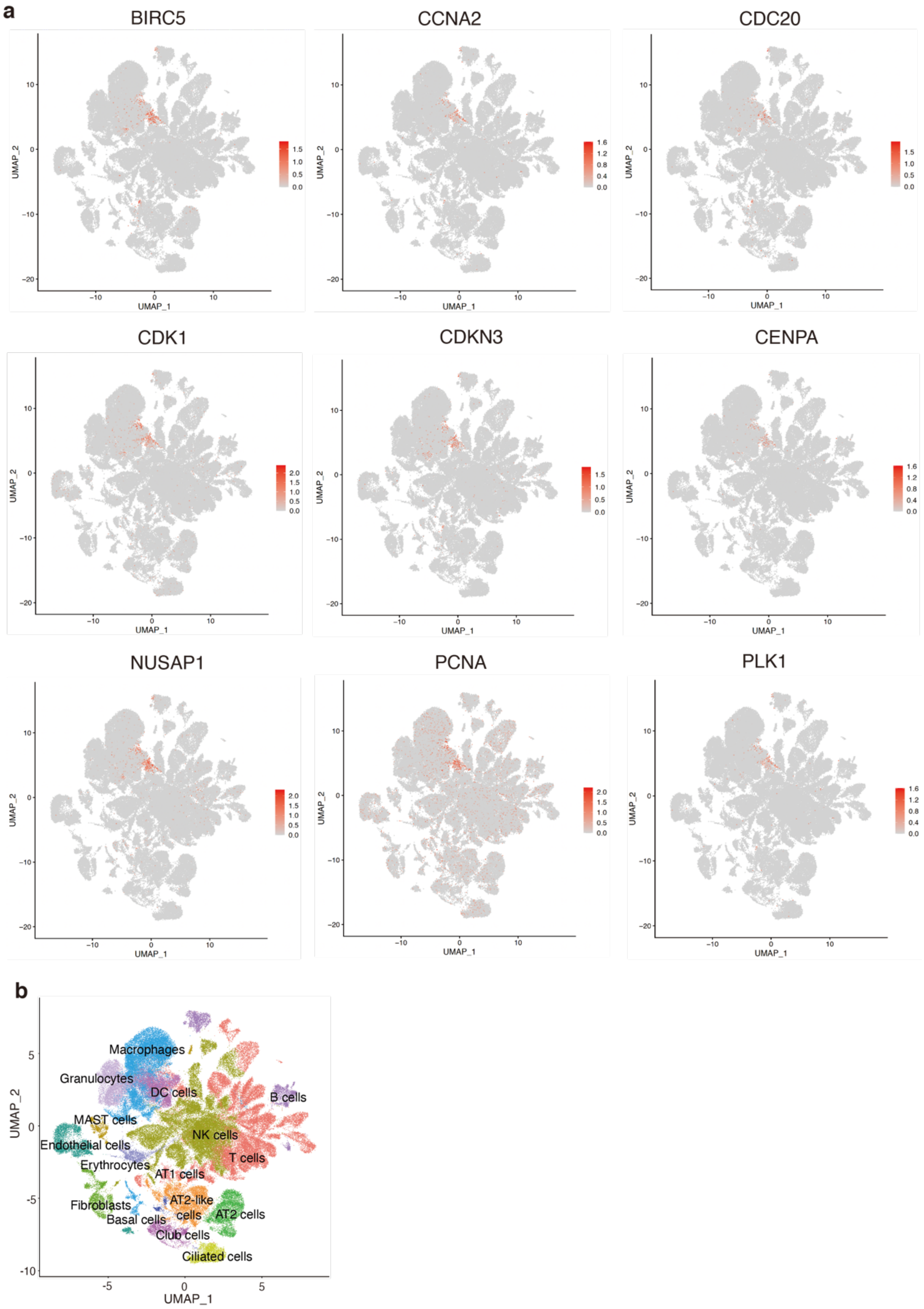

Fig. S6. Distinct cell proliferation markers and color-coded by their associated clusters. (a) Expression of cell proliferation markers in single-cell transcriptomic profiles. (b) UMAP visualization of the 16 major cell types identified, color-coded by their associated clusters.

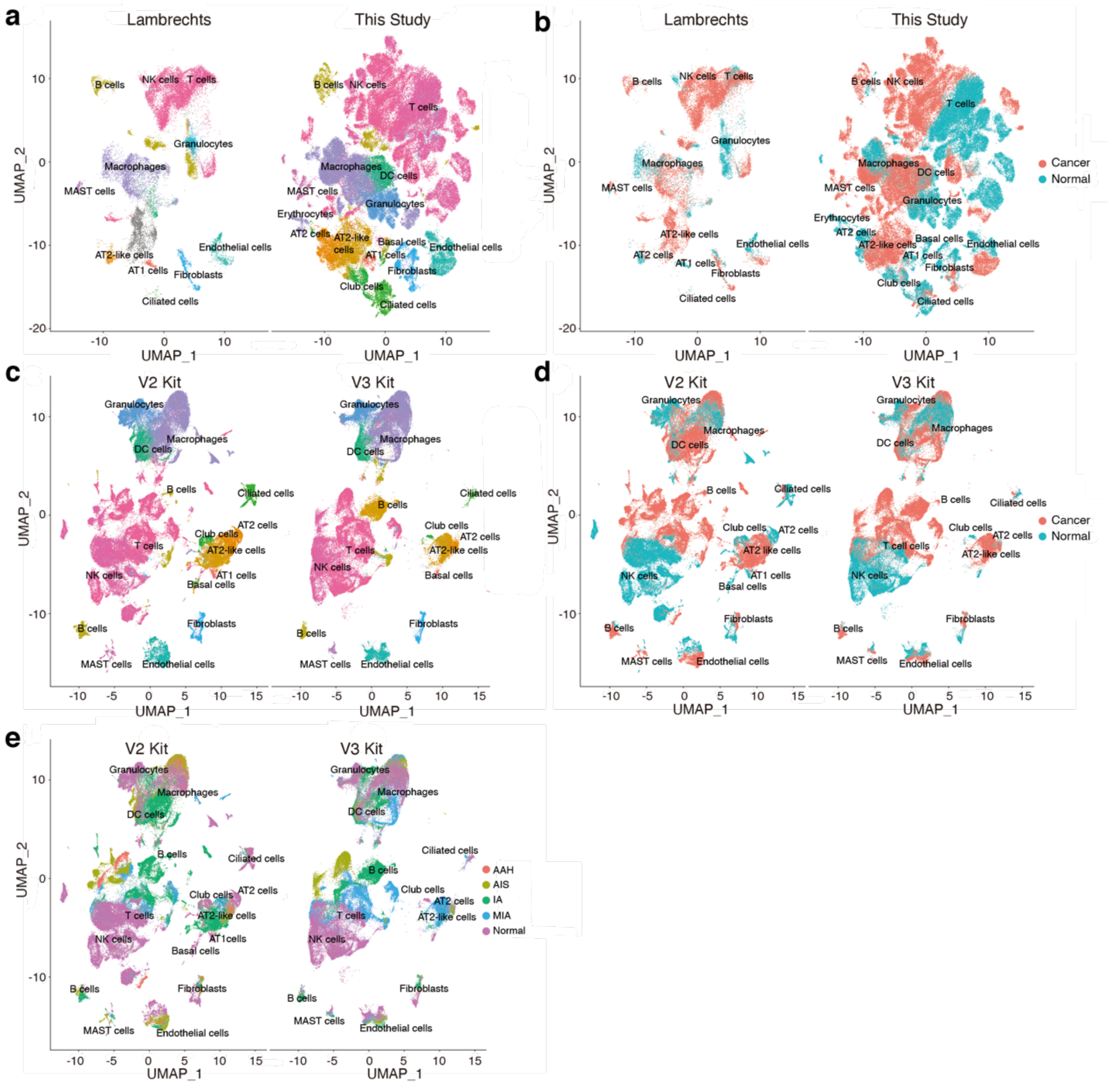

Fig. S7. Comparison of our data with data from Lambrecht et al. (a) Transcriptomic identification of major cell types identified in Lambrechts et al and our study, visualized by UMAP. Cancer cells from patients 6 and 7 were identified in the study by Lambrechts et al. (b) UMAP visualization of major cell types from tumor and normal tissues. (c). UMAP visualization of the major cell types identified from 52 samples processed by 10X Chromium V2 or V3 kits. (d) UMAP visualization of cell types from cancer and normal tissues processed by 10X Chromium V2 or V3 kits. (e) UMAP visualization of cell types in four histologic stages processed by 10X Chromium V2 or V3 kits.

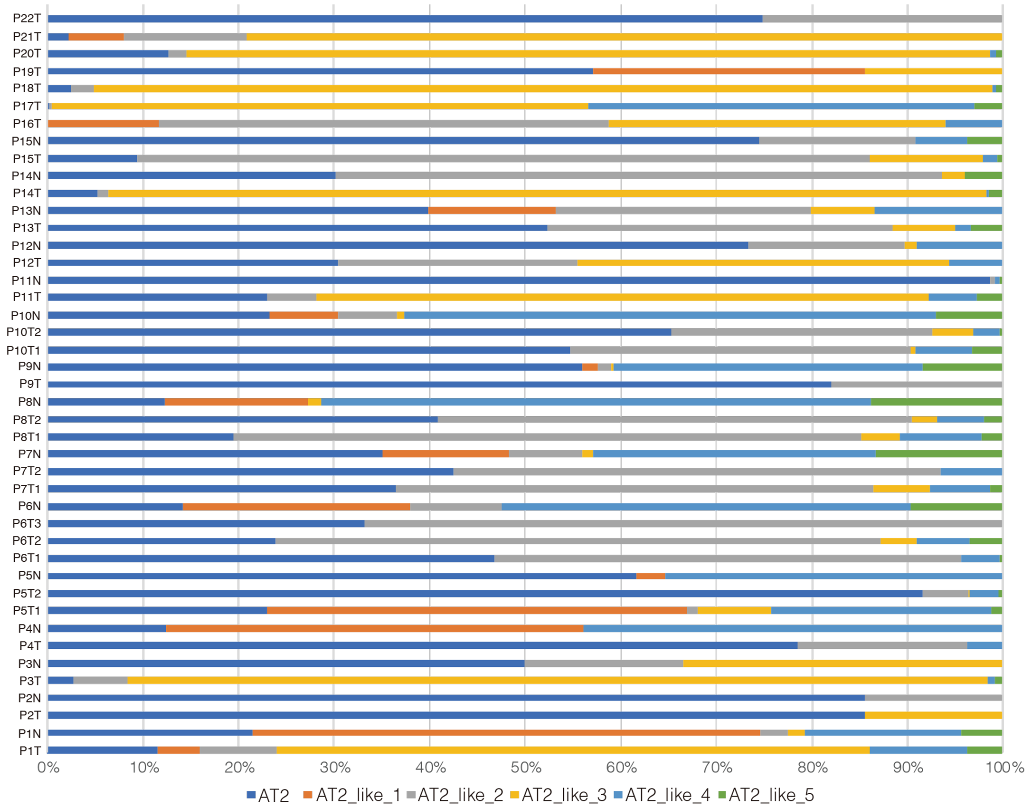

Fig. S8. Proportion of cells originating from different samples in the six major epithelial cell types. The labels indicate tumor identifiers. Source data are provided as a Source Data file.

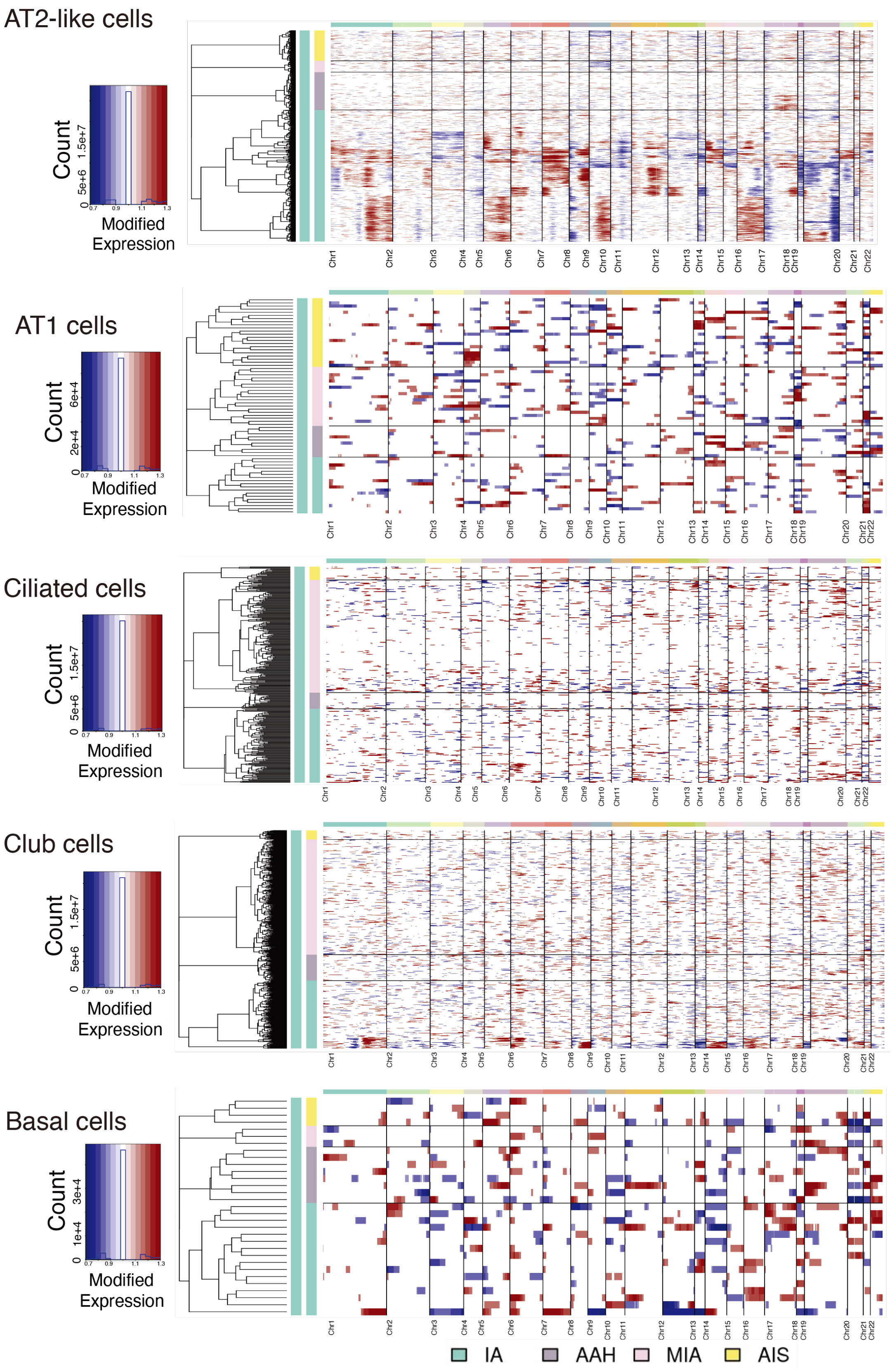

Fig. S9. Representative inferred CNV heatmaps with hierarchical clustering of AT2-like, AT1, ciliated, club, and basal cells.

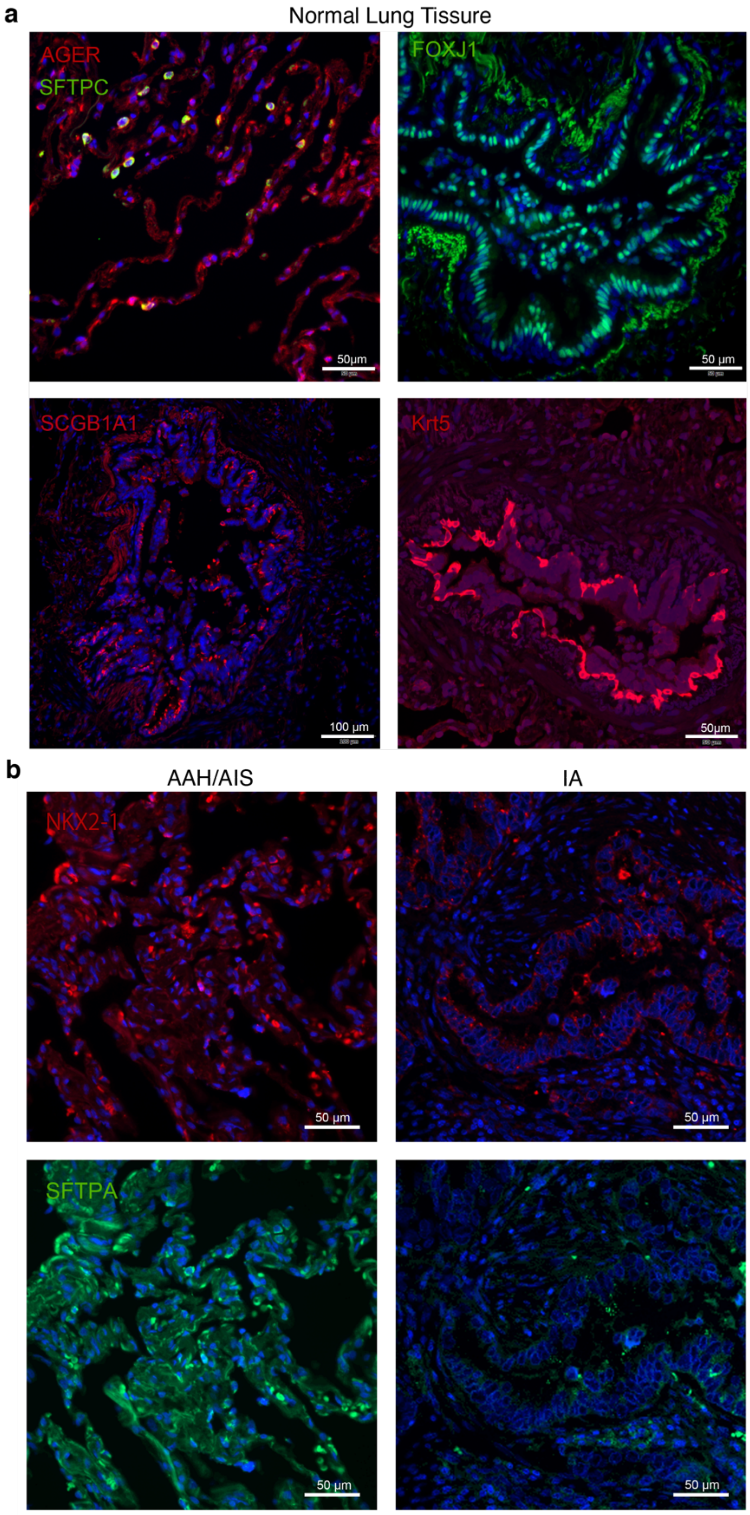

Fig. S10. Immunofluorescence confirmation of epithelial cell markers in normal and tumor samples. (a) Immunostaining protein markers for AT1 cells (AGER, red), AT2 cells (SFTPC, green), ciliated cells (FOXJ1, green), club cells (SCGB1A1, red) and basal cells (Krt5, red) in normal lung resections. (b) Immunostaining NKX2-1and SFTPA in different histologic stages of LUAD tissues. Each staining in panels come from three samples. Nuclei (DAPI) were stained blue. Scale bars: 50μm.

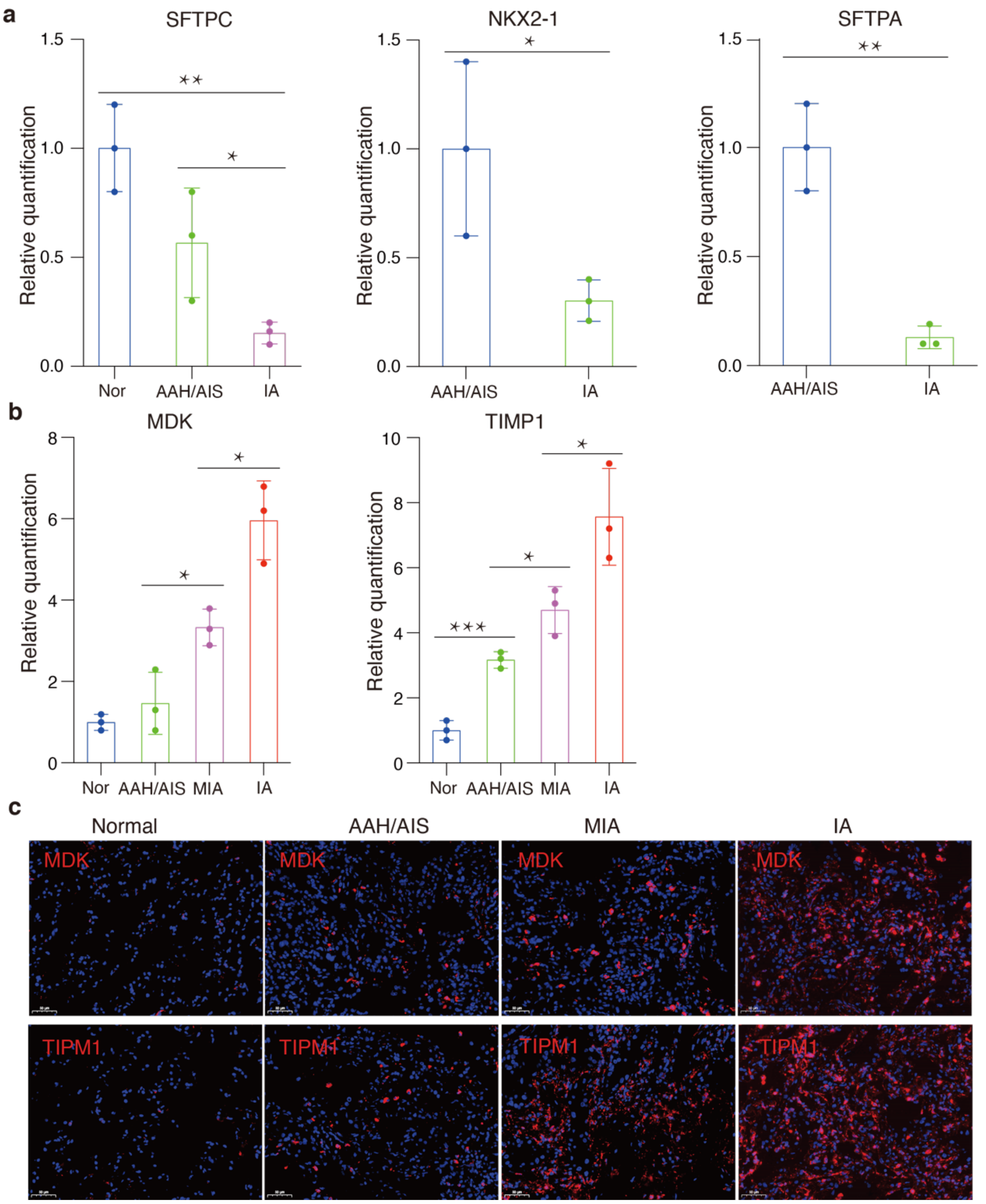

Fig. S11. Validation of gene expression features in human normal lung and LUAD tissues from different histologic stages. Quantification of relative expression of different genes staining in panel (a) are the shown in Fig. 2g and Fig. S10b (SFTPC: Nor vs IA p value is 0.00207, AAH/AIS vs IA p value is 0.0493; Nkx2-1: AAH/AIS vs IA p value is 0.0426; SFTPA: AAH/AIS vs IA p value is 0.00188), staining in panel (b) are the shown in Fig. S11c (MDK: AAH/AIS vs MIA p value is 0.0219, MIA vs IA p value is 0.0131; TIMP1: Nor vs AAH/AIS p value is 0.000662, AAH/AIS vs MIA p value is 0.0254; MIA vs IA p value is 0.0396); (c) Immunostaining of MDK (top) and TIMP1 (bottom) in normal lung and tumor tissues from different stages. (n = 3 replicates; *P < 0.05, **P < 0.01, ***P < 0.001). Unpaired two-tailed t-test was used to compare two groups. Error bars represent mean ± standard error of mean (s.e.m.). Source data are provided as a Source Data file.

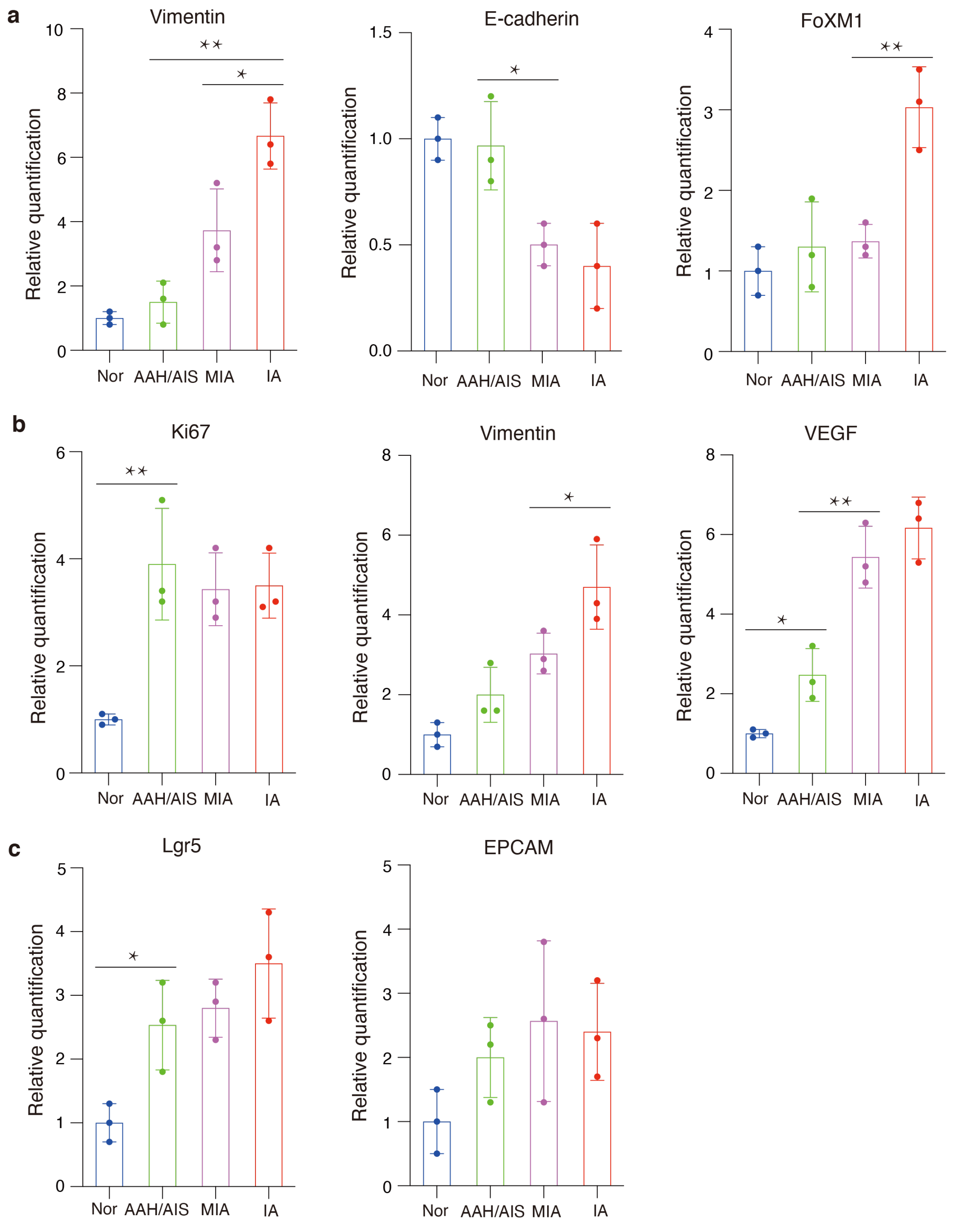

Fig. S12. Quantification of relative expression of different genes in normal lung and LUAD tissues from different histologic stages. Staining in panel (a) are the shown in Fig.3e (Vimentin: AAH/AIS vs IA p value is 0.00183, MIA vs IA p value is 0.0366; E-cadherin: AAH/AIS vs MIA p value is 0.0249; FoXM1: MIA vs IA p value is 0.00609); Staining in panel (b) are the shown in Fig. S13 (Ki67: Nor vs AAH/AIS p value is 0.00872; Vimentin: MIA vs IA p value is 0.0474; VEGF: Nor vs AAH/AIS p value is 0.0196, AAH/AIS vs MIA p value is 0.00737); Staining in panel (c) are the shown in Fig. S14 (Lgr5: Nor vs AAH/AIS p value is 0.0254). (n = 3 replicates; *P < 0.05, **P < 0.01, ***P < 0.001). Unpaired two-tailed t-test was used to compare two groups. Error bars represent mean ± standard error of mean (s.e.m.). Source data are provided as a Source Data file.

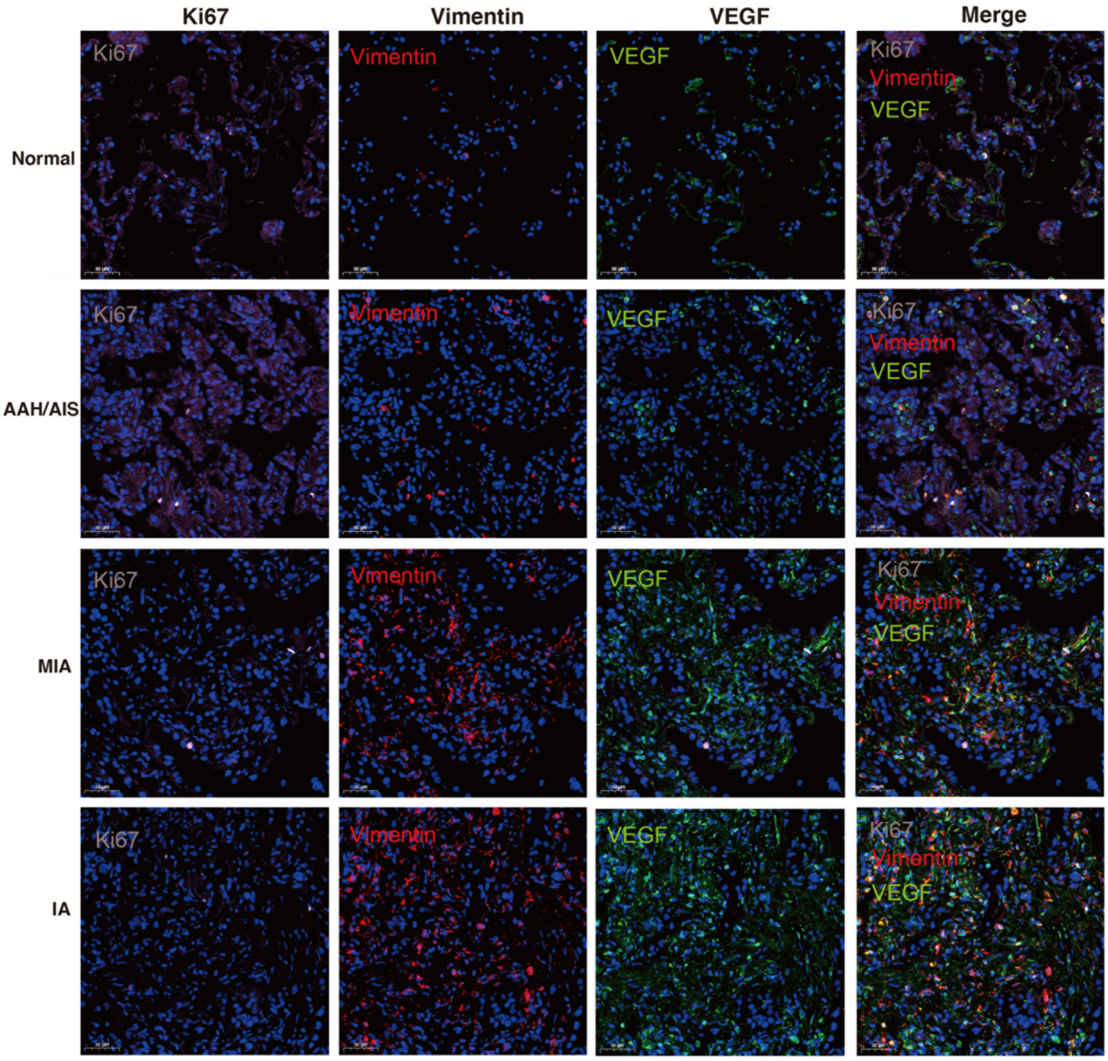

Fig. S13. Multiplexed immunostaining of Ki67 (cell growth, gray), Vimentin (EMT, red), and VEGF (angiogenesis, green) in normal and LUAD tissues from different histologic stages.. Each staining in panels come from three samples. Nuclei (DAPI) were stained blue. Scale bars: 50μm.

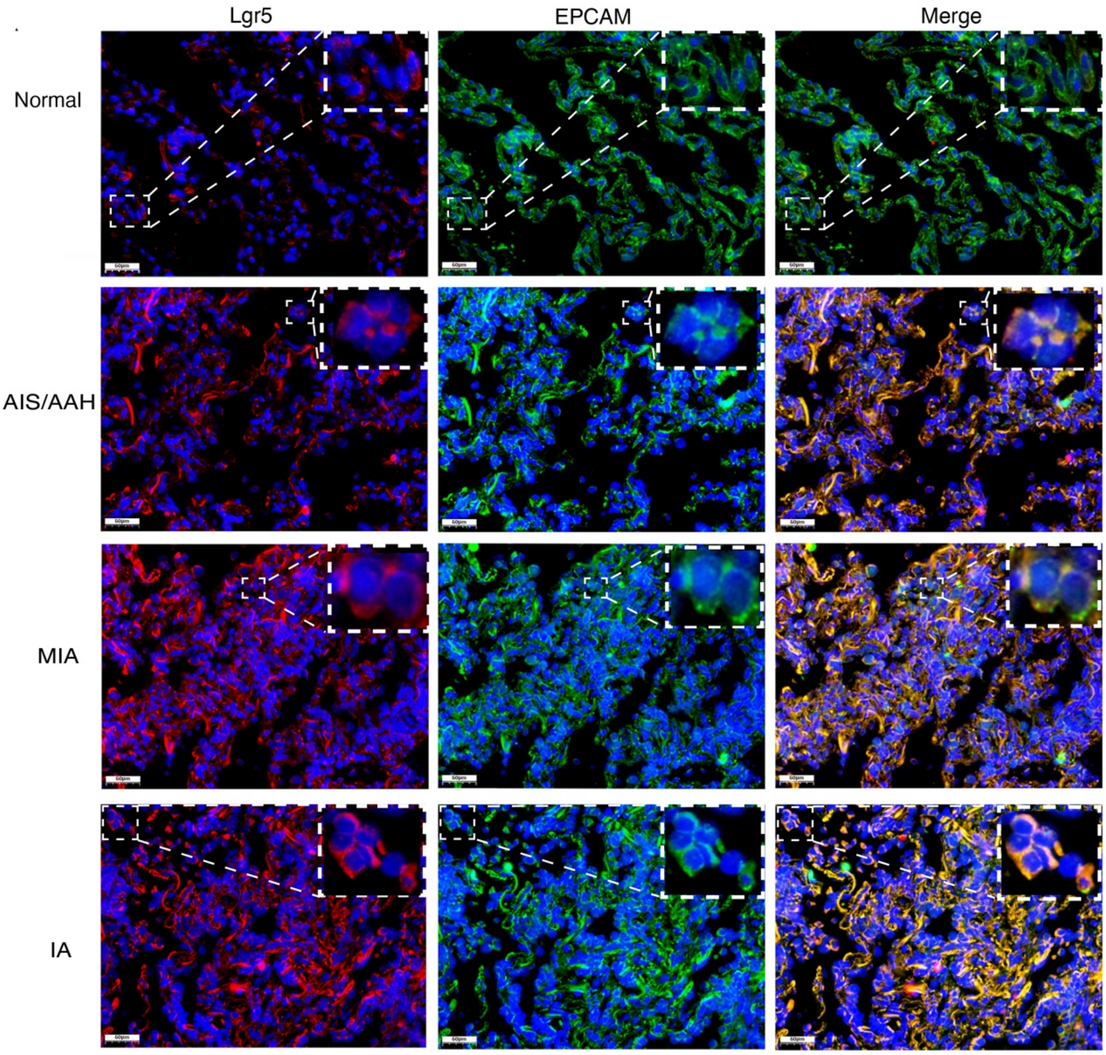

Fig. S14. Multiplexed fluorescent in situ hybridization staining of mRNA of Lgr5 (red) or EPCAM (green) in normal tissues and LUAD tissues from different histologic stages. Each staining in panels come from three samples. Nuclei (DAPI) were stained blue. Scale bars: 50μm.

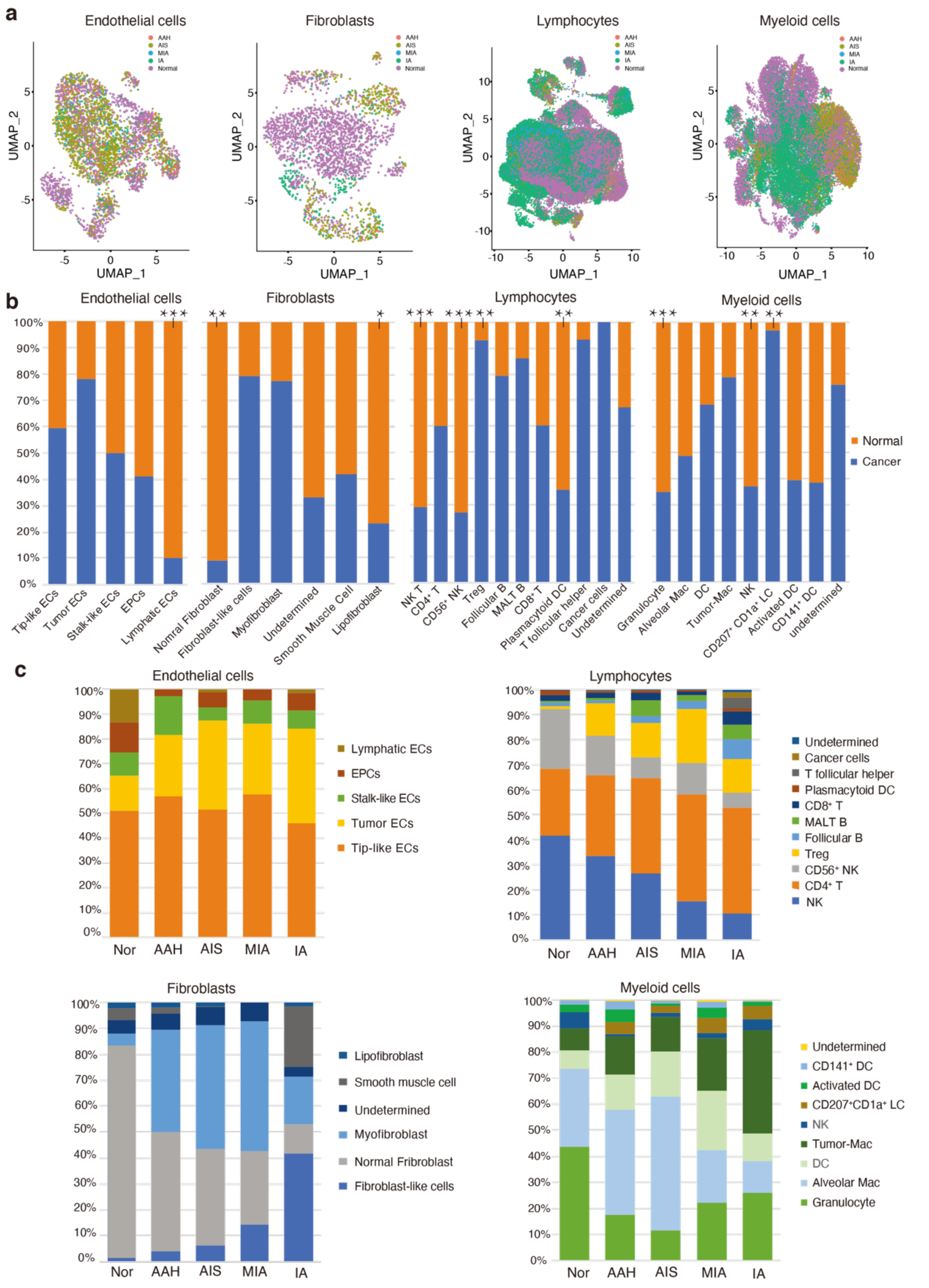

Fig. S15. Identification and characterization of stromal and immune cell subtypes. (a) UMAP visualization of stromal and immune cells color-coded by histologic stages. (b) Proportions of cells originating from different sample types in the cluster subtypes (Lymphatic ECs: P=0.000578, Normal fibroblasts: P=0.00255, Lipofibroblasts: P=0.0163, NK T cells: P= 1.62e-05, $CD56^{+}$ NK cells: P= 2.55e-05, Treg cells: P=0.00645, Plasmacytoid DC cells: P=0.00360, Granulocytes: P= 3.50037e-05, NK cells: P=0.00831, $CD207^{+}CD1a^{+}$ LC cells: P=0.00564; *P < 0.05, **P < 0.01, ***P < 0.001). Unpaired two-tailed t-test was used to compare two groups. (c) Percentages of endothelial cells, fibroblasts, lymphocytes, and myeloid cells in their respective clusters by histologic stage. Source data are provided as a Source Data file.

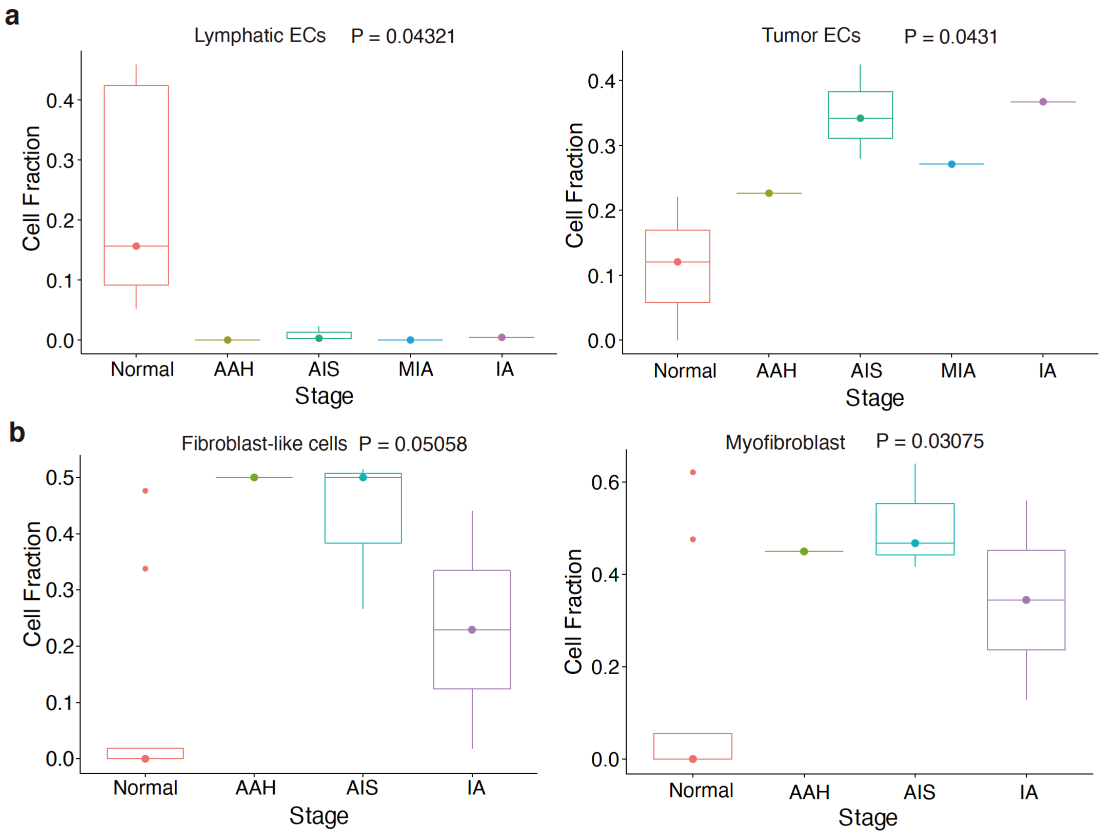

Fig. S16. Proportion of endothelial cells and fibroblasts varied among normal lung and tissues from AAH AIS, MIA and IA stage. (a) Proportion of endothelial cell types varied among normal lung tissues (n=7 replicates) and tissues from AAH (n=1), AIS (n=3), MIA (n=1) to IA (n=1). (b) Proportion of fibroblast types varied among normal lung tissues (n=9 replicates) and tissues from AAH (n=1), AIS (n=3) to IA (n=2). Differences were assessed for significance using the Kruskal-Wallis rank test, one-sided test. Center line: Median; Box limits: Upper and lower quartiles. Source data are provided as a Source Data file.

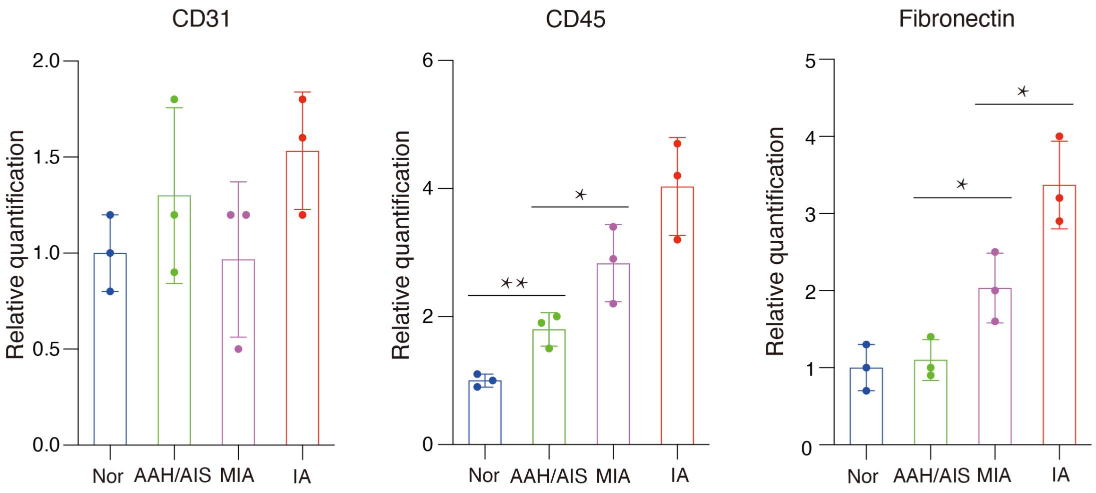

Fig. S17. Quantification of relative expression of different genes in normal lung tissues and LUAD tissues from different histologic stages. Staining in panel are the shown in Fig.4k (CD45: Nor vs AAH/AIS p value is 0.00805, AAH/AIS vs MIA p value is 0.0390; Fibronectin: AAH/AIS vs MIA p value is 0.0365, MIA vs IA p value is 0.0335). (n = 3 replicates; *P < 0.05, **P < 0.01, ***P < 0.001). Unpaired two-tailed t-test was used to compare two groups. Error bars represent mean ± standard error of mean (s.e.m.). Source data are provided as a Source Data file.

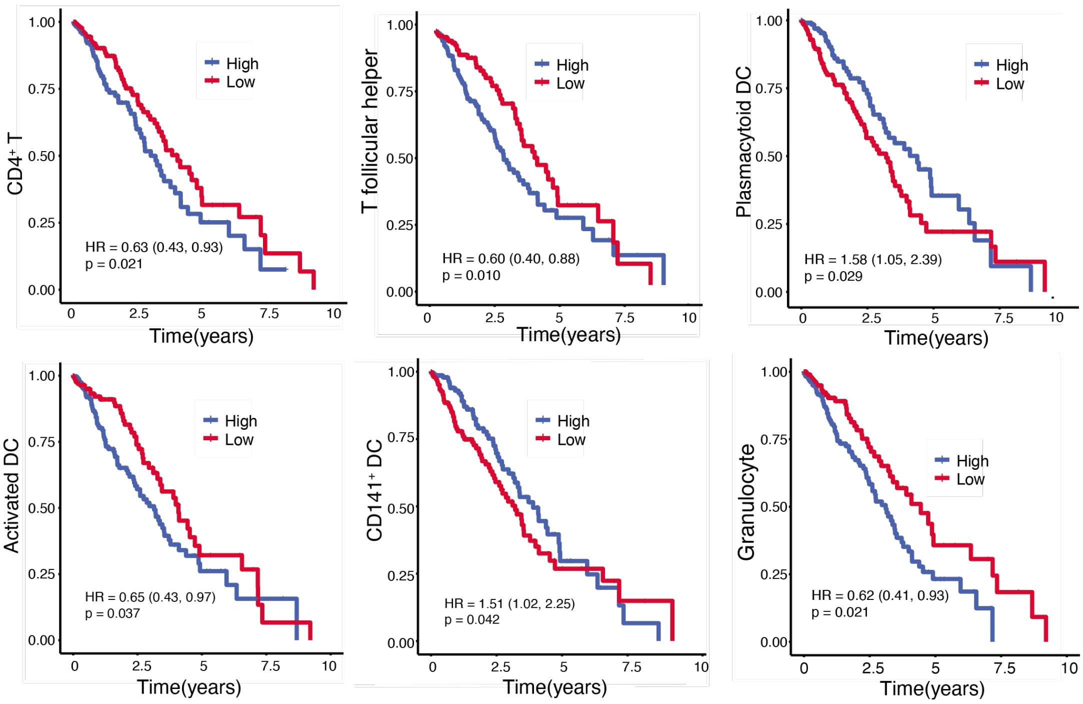

Fig. S18. Association between gene expression in LUAD subclusters and patient survival. Kaplan–Meier survival curves for patients with LUAD (n=515) were stratified by high or low expression of marker genes (based on the observed median expression levels) in the indicated cell subtypes among all patients. Hazard ratios (HRs) are indicated with their 95% confidence intervals in brackets. The indicated Cox regression P values were obtained after correcting for age, sex, and tumor stage.

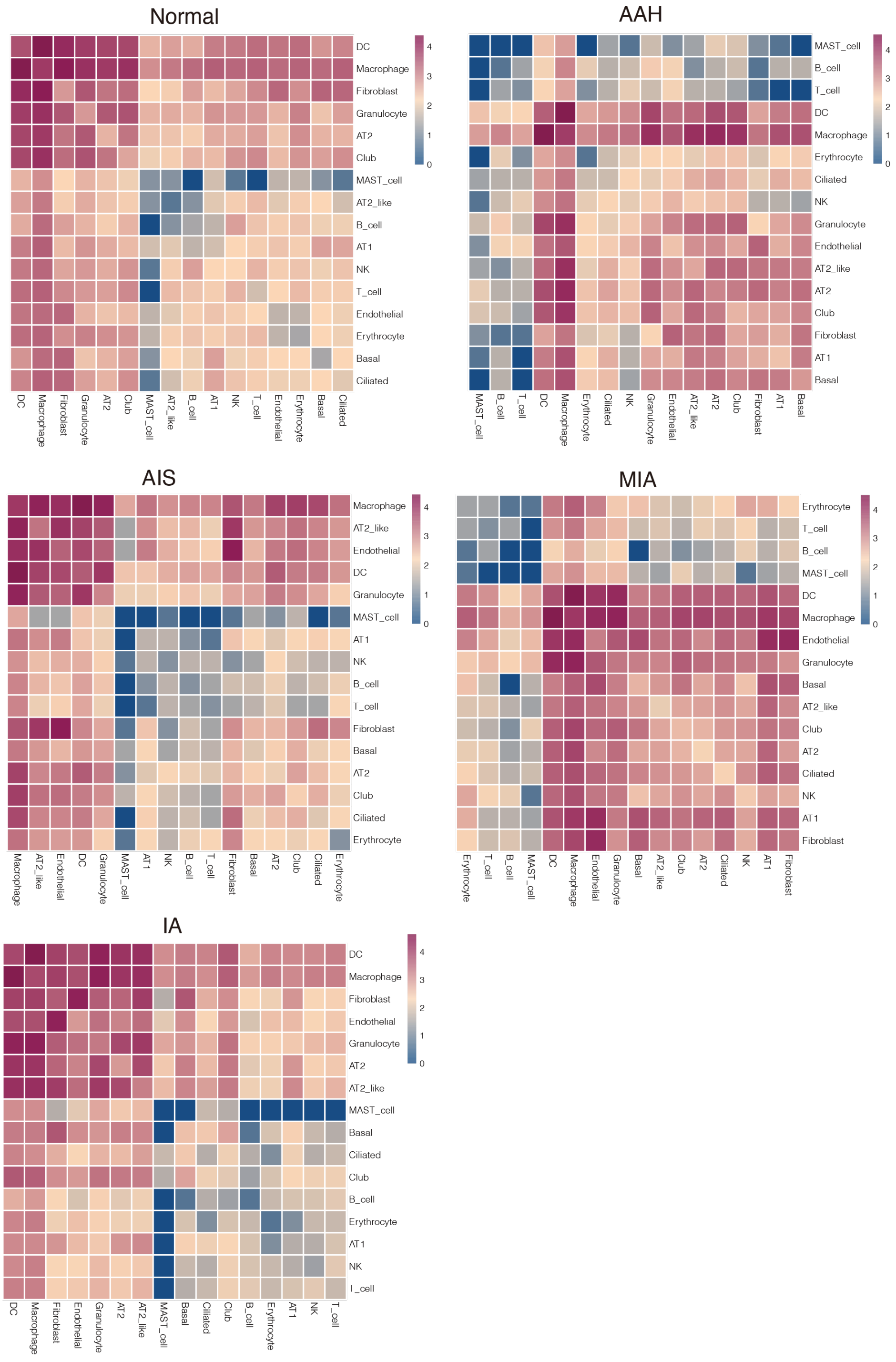

Fig. S19. Heat map depicting the significant interactions among the 16 major cell types in normal lung tissues and LUAD tissues from different histologic stages.

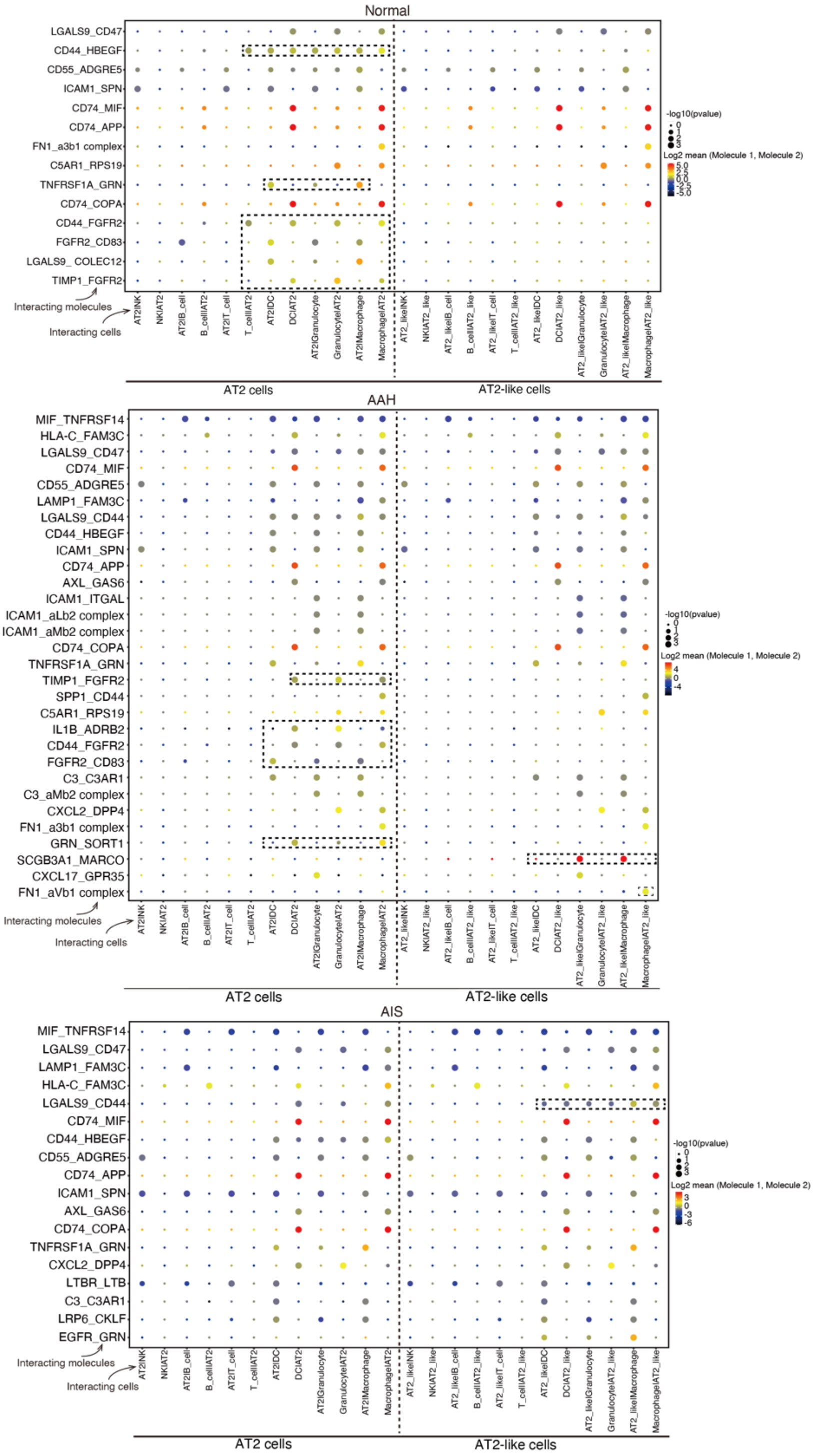

Fig. S20. Selected ligand-receptor interactions in normal lung tissues and tissues from AAH to AIS. P values (two-tailed permutation test) are indicated by circle size; the scale is on the right. The means of the average expression level of interacting molecule 1 in cluster 1 and interacting molecule 2 in cluster 2 are indicated by color. Assays were carried out at the RNA level, but extrapolated to protein interactions. Selected cells include AT2 cells and AT2-like cells.

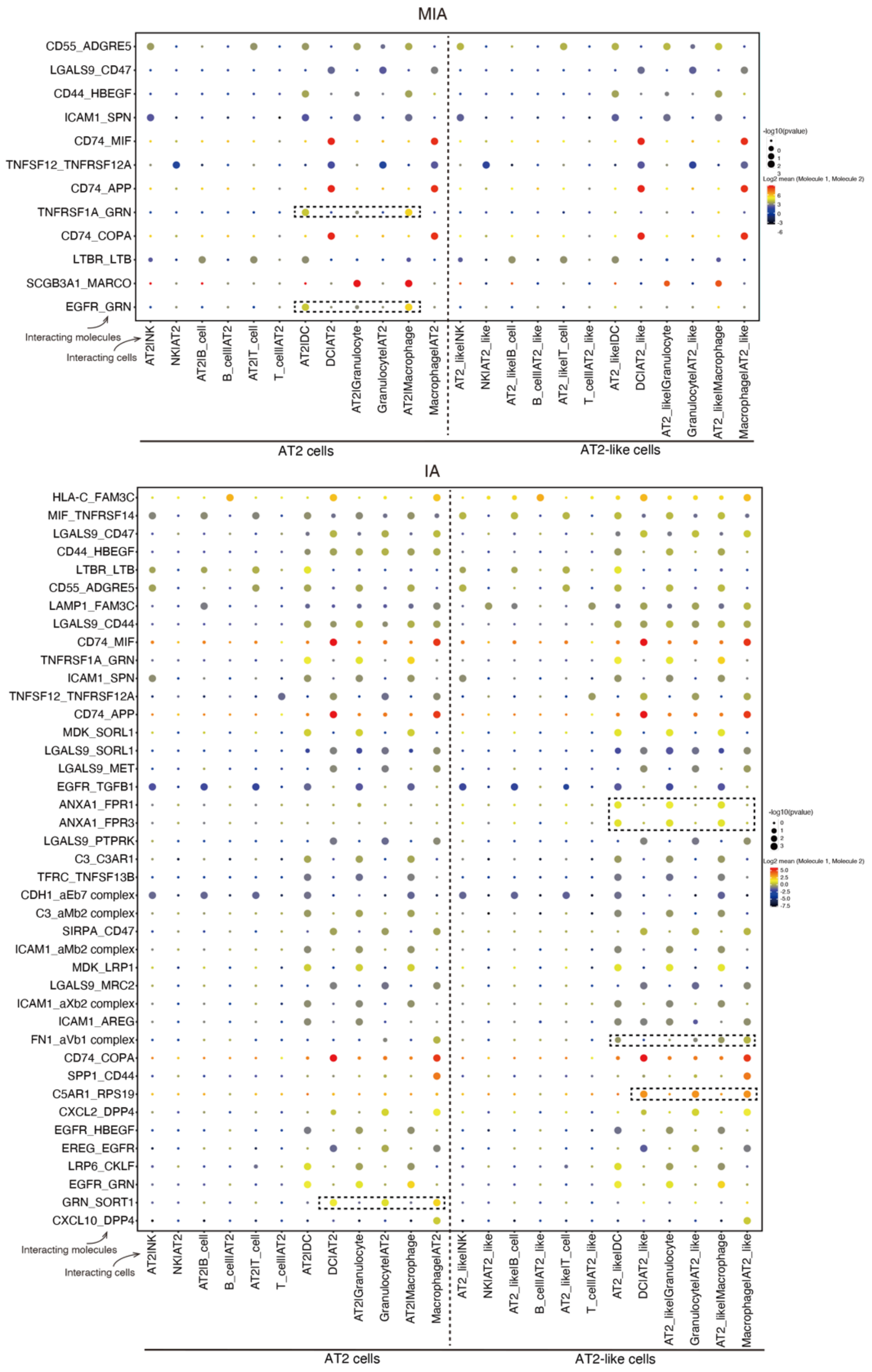

Fig. S21. Selected ligand-receptor interactions in tissues from MIA to IA. P values (two-tailed permutation test) are indicated by circle size; the scale is on the right. The means of the average expression level of interacting molecule 1 in cluster 1 and interacting molecule 2 in cluster 2 are indicated by color. Assays were carried out at the RNA level, but extrapolated to protein interactions. Selected cells include AT2 cells and AT2-like cells.

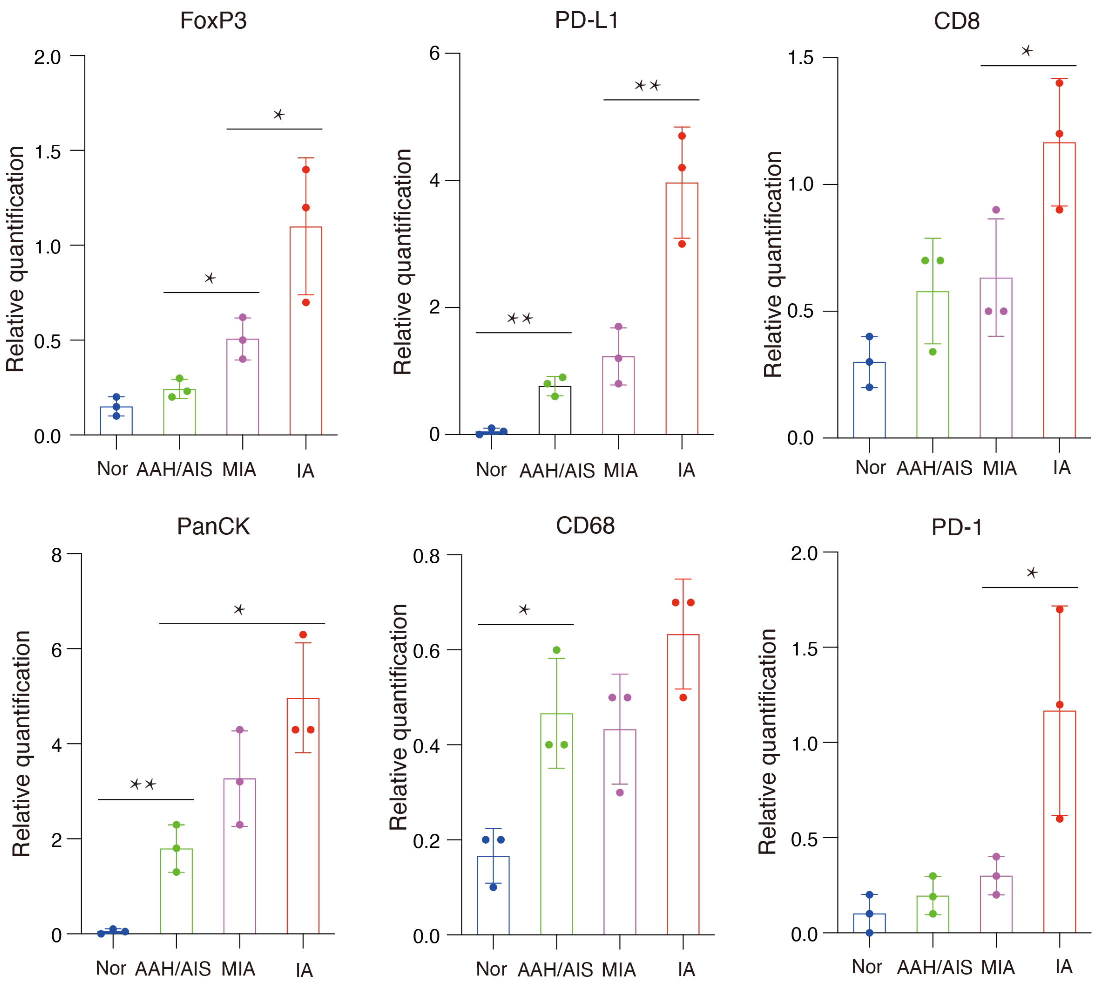

Fig. S22. Quantification of relative expression of different genes in normal lung and LUAD tissues from different histologic stages (FoxP3: AAH/AIS vs MIA p value is 0.0199, MIA vs IA p value is 0.0288; PD-L1: Nor vs AAH/AIS p value is 0.00151, MIA vs IA p value is 0.00855; CD8: MIA vs IA p value is 0.0325; PanCK: Nor vs AAH/AIS p value is 0.00381, AAH/AIS vs IA p value is 0.0121; CD68: Nor vs AAH/AIS p value is 0.0158; PD-1: MIA vs IA p value is 0.0378). Staining in panel are the shown in Fig. 5d (n = 3 replicates; *P < 0.05, **P < 0.01). Unpaired two-tailed t-test was used to compare two groups. Error bars represent mean ± standard error of mean (s.e.m.). Source data are provided as a Source Data file.

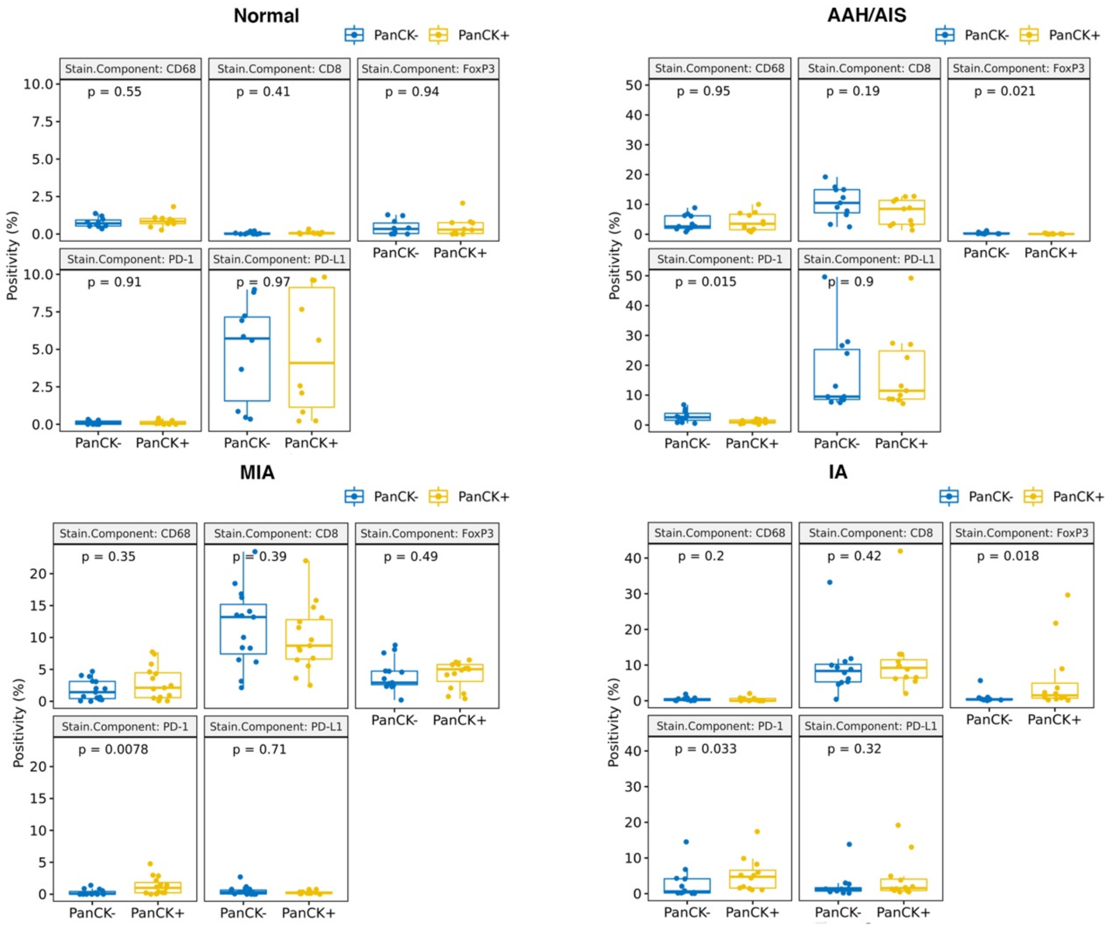

Fig. S23. Quantification of the relative fluorescence intensity of cell types based on PanCK$^-$ and PanCK$^+$ from normal to IA stage LUAD in Fig 5d. The area and percentage signal intensity are calculated for each tissue (n=3 replicates), The median is shown by the centre line in the box. The first quartile marks one end of the box and the third quartile marks the other end of the box. The whiskers extend from the ends of the box to the smallest and largest data values. Two-sided Wilcoxon test was used to compare the positivity between two groups. Error bars represent mean ± standard error of mean (s.e.m.). Source data are provided as a Source Data file.

Table S1. FISH probes and antigens targeted by the antibodies that were used to detect the fluorescence on the normal and lung cancer tissues.

FISH Probe	Description
AT2-like Markers	
Lgr5	Stem Cells
Vimentin	Mesenchymal Cells
E-cadherin	Cell Adhesion
FOXM1	Stem Cells
EPCAM	Epithelial Cells

Antigen	Description
Epithelial Markers	
SFTPC	AT2 Cells
SCGB1A1	Club Cells
AGER	AT1 Cells
FOXJ1	Ciliated Cells
EPCAM	Epithelial Cells
Nkx2-1	AT2 Cells
SFTPA	AT2 Cells

Antigen	Description
Stromal Context	
CD45	Pan-Immune
CD31	Endothelial cells
VEGF	Endothelial cells

Antigen	Description
Lung Cancer Multiplex Markers	
FoxP3	Regulatory T cells
Cytokeratin	Tumor cells
CD8	Cytotoxic T cells
CD68	Macrophages
EPCAM	Epithelial Cells
PD-1	Check point markers
PD-L1	Check point markers

Antigen	Description
Cell Growth	
Ki67	Proliferation
MDK	Progression
TIMP1	Progression

Antigen	Description
Mesenchymal Markers	
Vimentin	Mesenchymal Cells
E-cadherin	Cell Adhesion
Fibronectin	Matrix Glycoprotein
