# Supplementary material for: Deciphering cell lineage specification of human lung adenocarcinoma with single-cell RNA sequencing
Source: Nat Commun. 2021 Nov 11;12:6500. doi: 10.1038/s41467-021-26770-2 (PMC8586023; doi:10.1038/s41467-021-26770-2)
Supplement: Supplementary file 4 — Supplementary Data File 2 [file 41467_2021_26770_MOESM4_ESM.pdf]

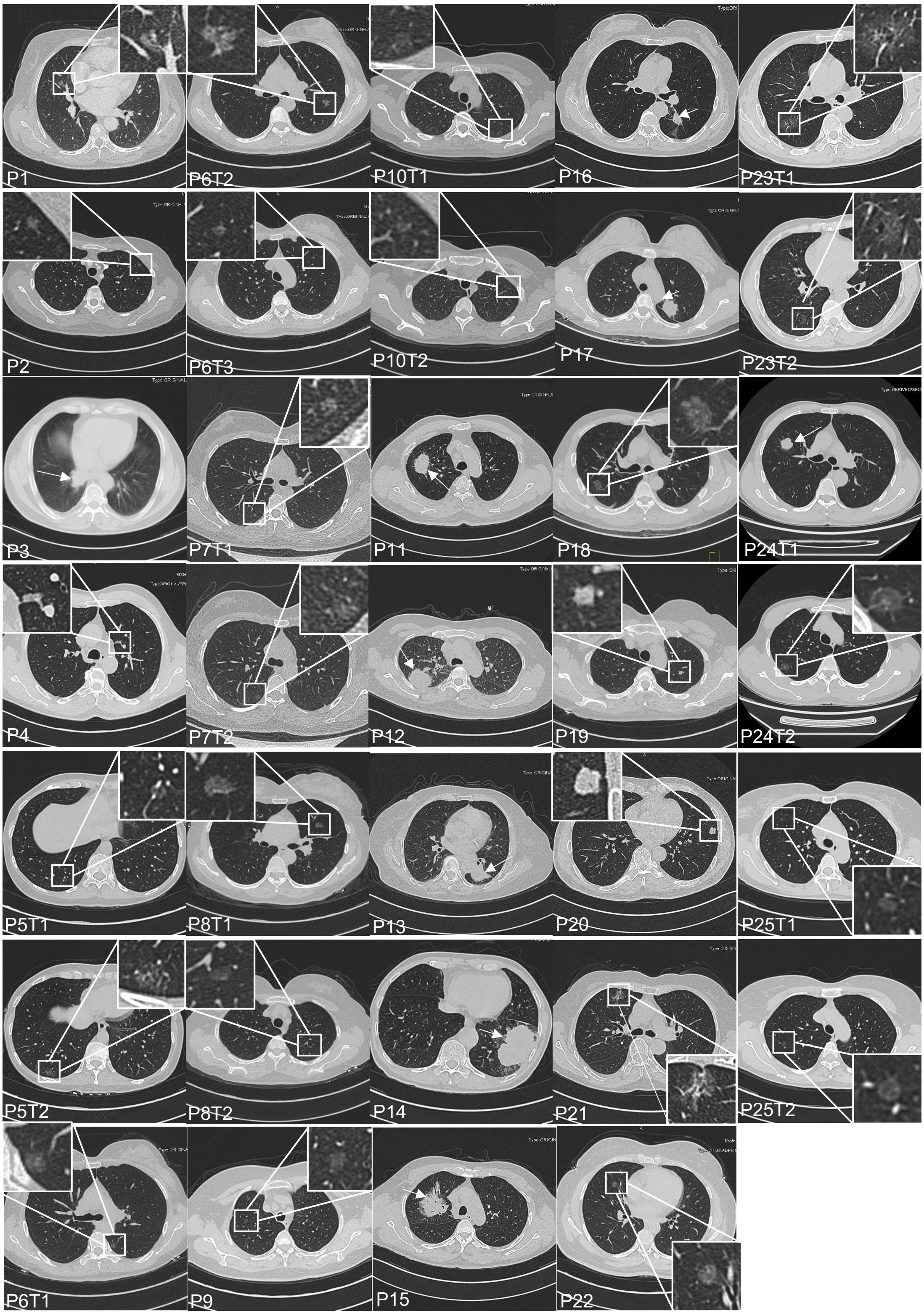

Supplementary Data File 2. CT scan images of all resected lung tumor specimens. A total of 34 resected lung tumor specimens were collected from 25 patients. All tumors showed pure ground glass opacity or a pure solid appearance, reducing the possibility of heterogeneity between sequencing samples and paraffin-embedded samples. The labels indicate tumor identifiers.
