# Supplementary material for: Deciphering cell lineage specification of human lung adenocarcinoma with single-cell RNA sequencing
Source: Nat Commun. 2021 Nov 11;12:6500. doi: 10.1038/s41467-021-26770-2 (PMC8586023; doi:10.1038/s41467-021-26770-2)
Supplement: Supplementary file 5 — Supplementary Data File 3 [file 41467_2021_26770_MOESM5_ESM.pdf]

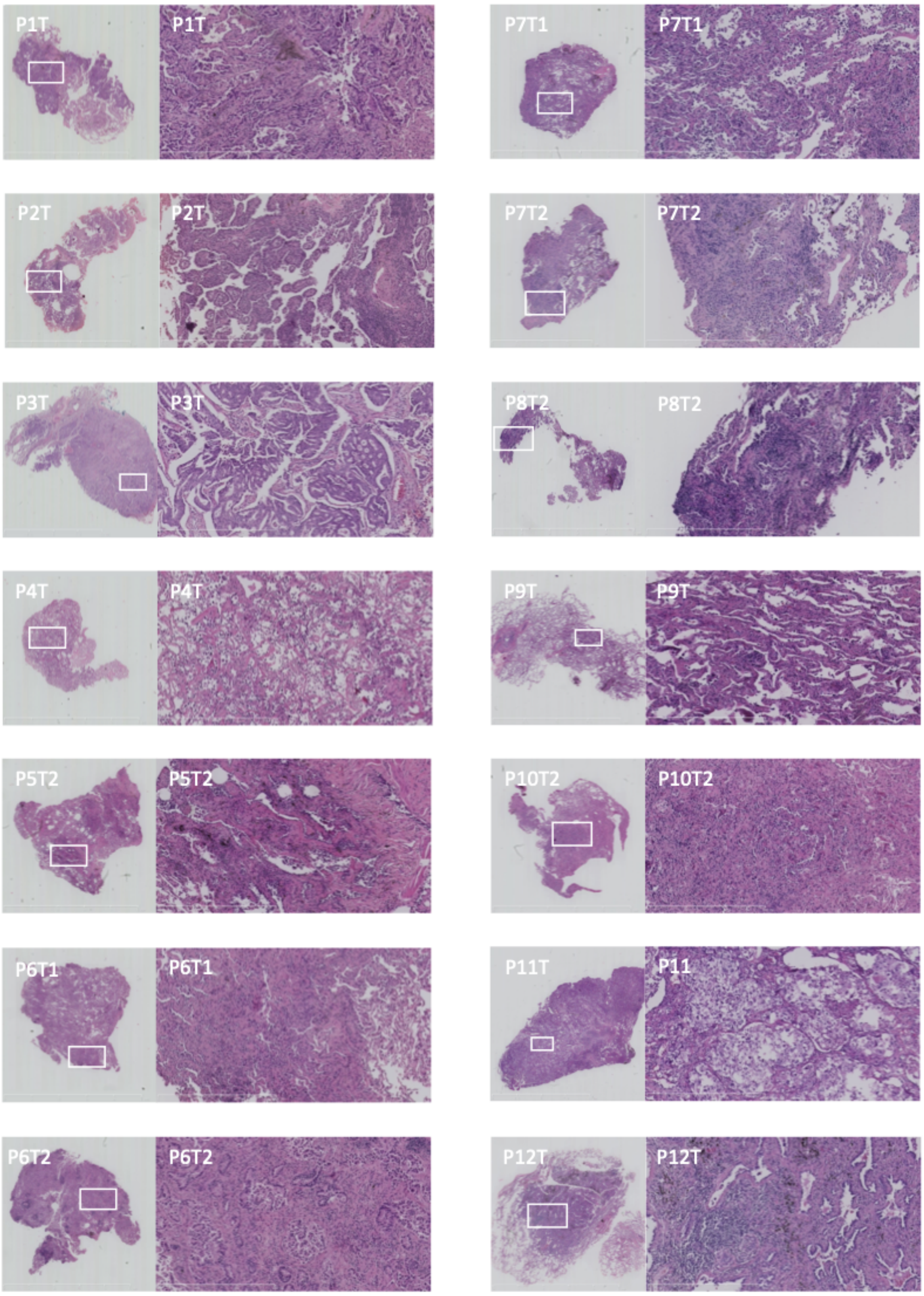
P1T
P1T
P7T1
P7T1
P2T
P2T
P7T2
P7T2
P3T
P3T
P8T2
P8T2
P4T
P4T
P9T
P9T
P5T2
P5T2
P10T2
P10T2
P6T1
P6T1
P11T
P11
P6T2
P6T2
P12T
P12T

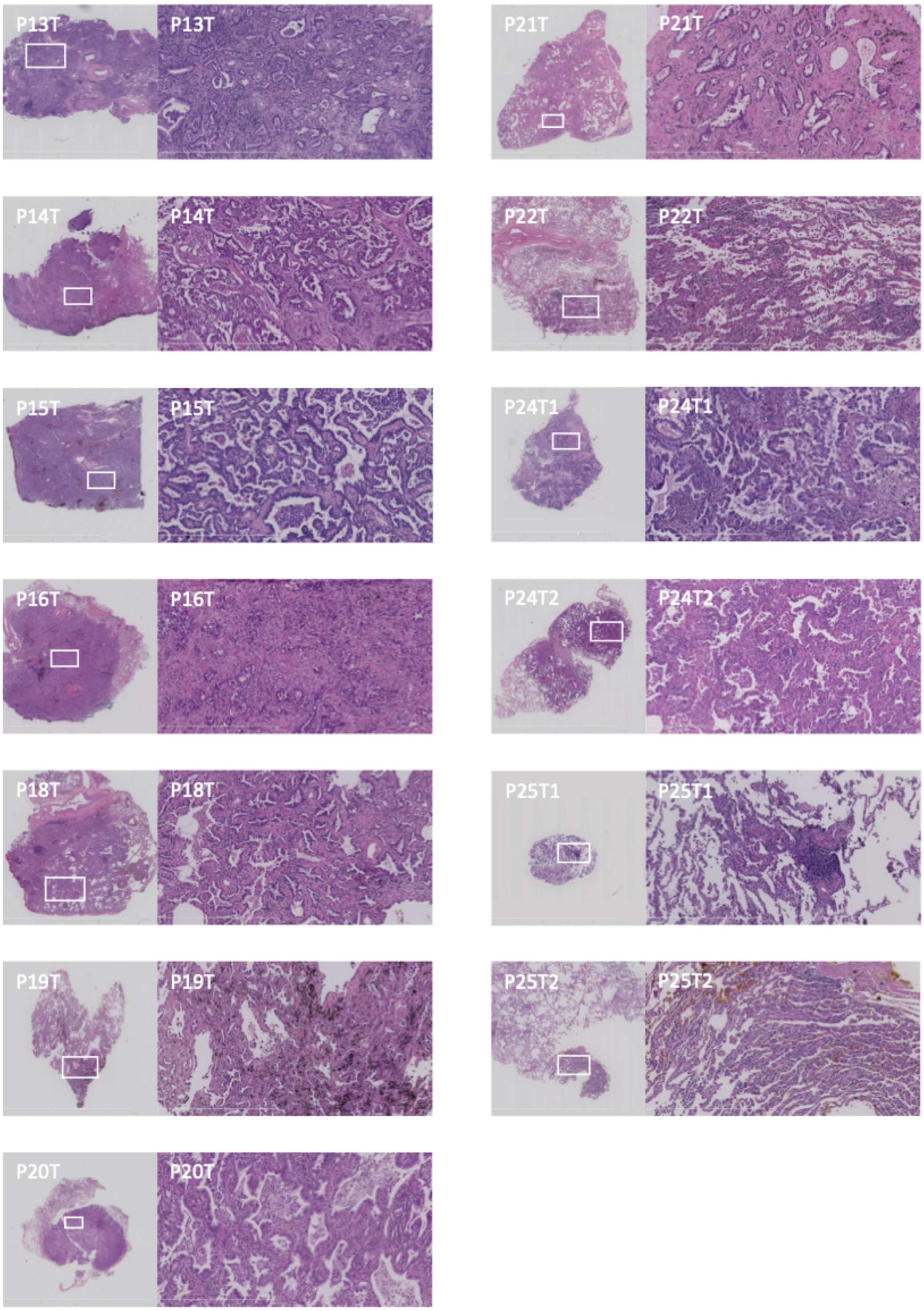

Supplementary Data File 3. H&E staining of paraffin-embedded sections cut from the tumors. Each staining was repeated three times independently on consecutive sections. The labels indicate tumor identifiers. Scale bars: 200µm.
