# Supplementary material for: Deciphering cell lineage specification of human lung adenocarcinoma with single-cell RNA sequencing
Source: Nat Commun. 2021 Nov 11;12:6500. doi: 10.1038/s41467-021-26770-2 (PMC8586023; doi:10.1038/s41467-021-26770-2)
Supplement: Supplementary file 11 — Supplementary Data File 9 [file 41467_2021_26770_MOESM11_ESM.pdf]

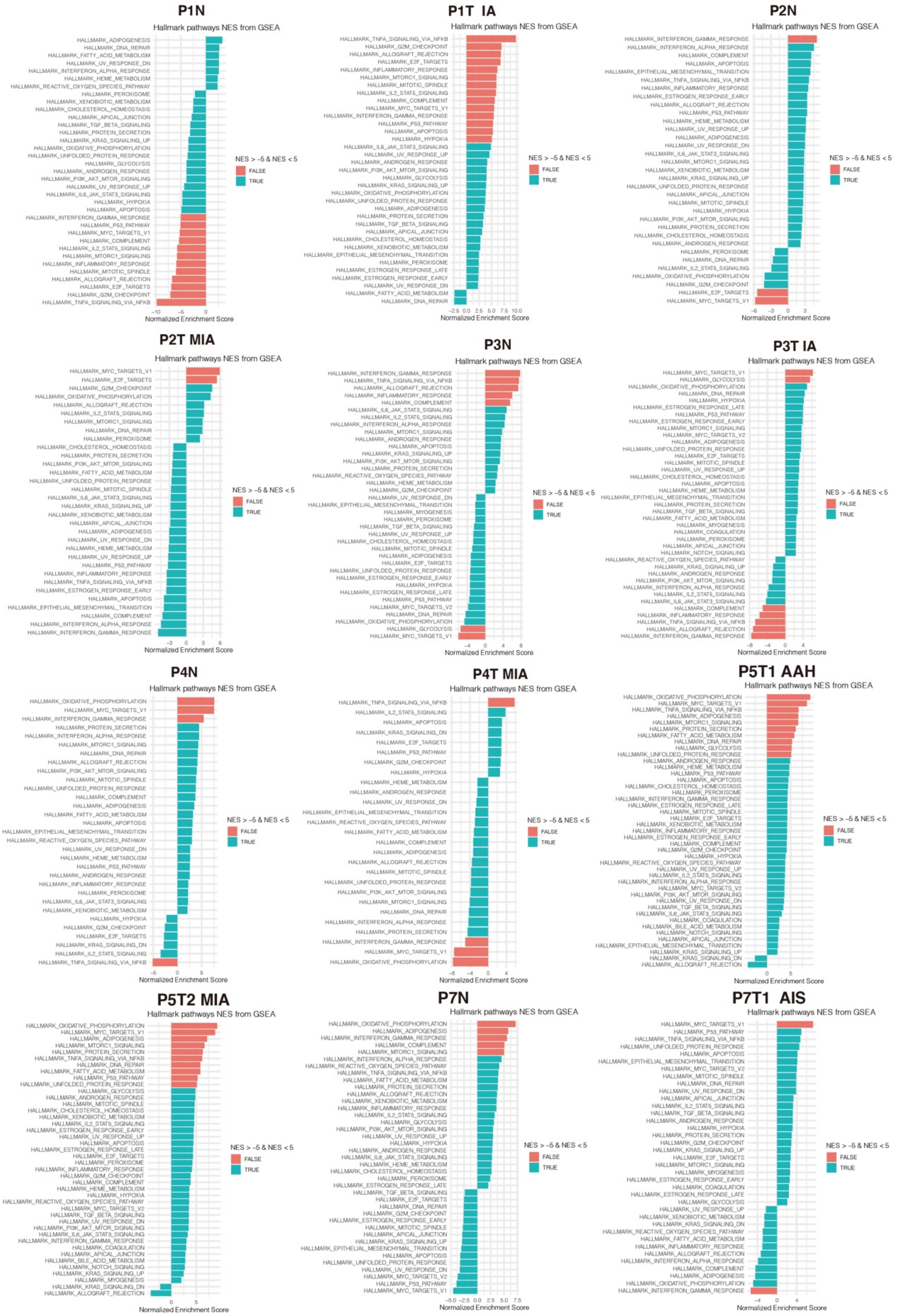
P1N
P1T IA
P2N
P2T MIA
P3N
P3T IA
P4N
P4T MIA
P5T1 AAH
P5T2 MIA
P7N
P7T1 AIS
Hallmark pathways NES from GSEA
NES > -5 & NES < 5
FALSE
TRUE
Normalized Enrichment Score

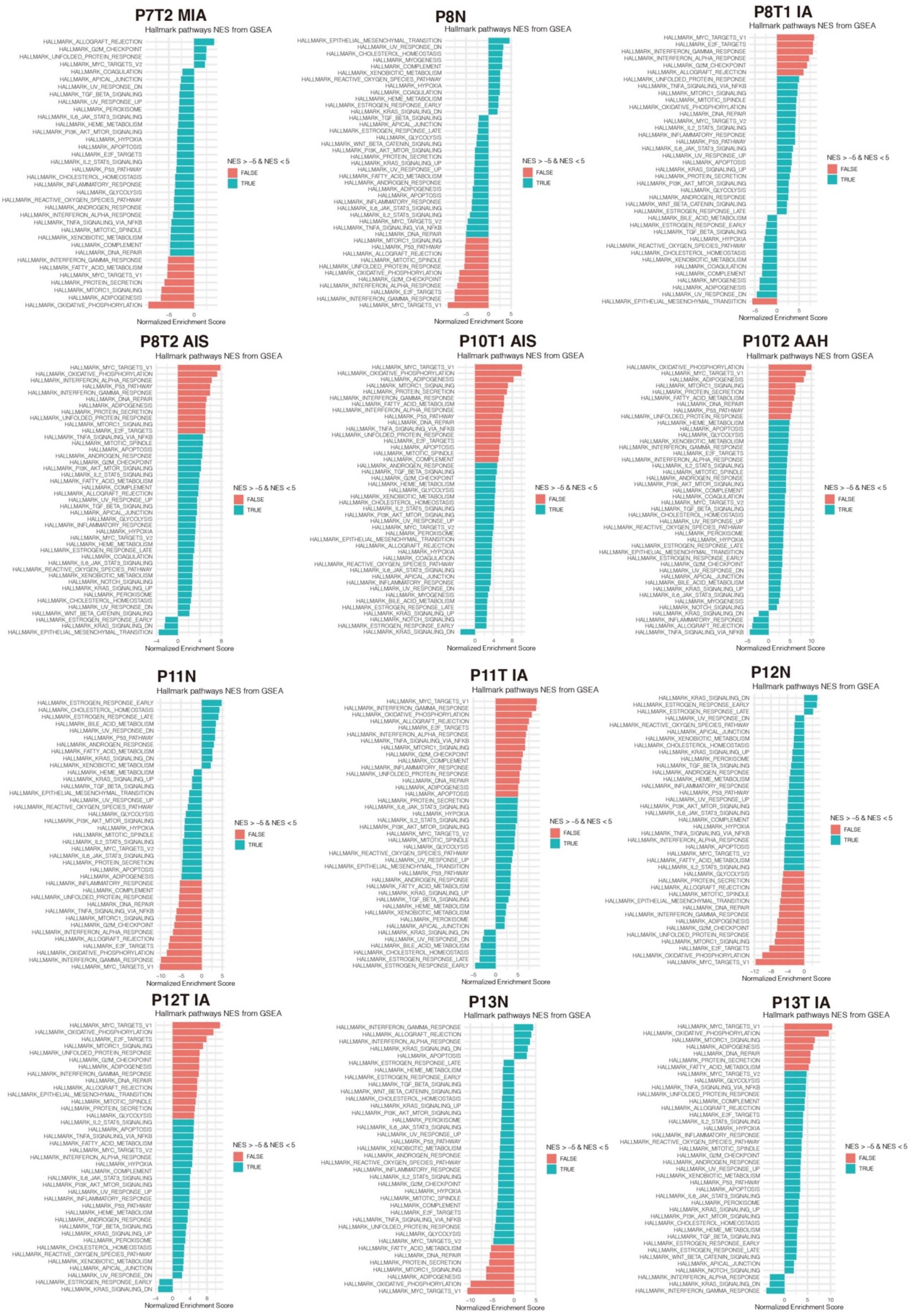
P7T2 MIA
Hallmark pathways NES from GSEA
Normalized Enrichment Score
NES > -5 & NES < 5
FALSE
TRUE
P8N
Hallmark pathways NES from GSEA
Normalized Enrichment Score
P8T1 IA
Hallmark pathways NES from GSEA
Normalized Enrichment Score
P8T2 AIS
Hallmark pathways NES from GSEA
Normalized Enrichment Score
P10T1 AIS
Hallmark pathways NES from GSEA
Normalized Enrichment Score
P10T2 AAH
Hallmark pathways NES from GSEA
Normalized Enrichment Score
P11N
Hallmark pathways NES from GSEA
Normalized Enrichment Score
P11T IA
Hallmark pathways NES from GSEA
Normalized Enrichment Score
P12N
Hallmark pathways NES from GSEA
Normalized Enrichment Score
P12T IA
Hallmark pathways NES from GSEA
Normalized Enrichment Score
P13N
Hallmark pathways NES from GSEA
Normalized Enrichment Score
P13T IA
Hallmark pathways NES from GSEA
Normalized Enrichment Score

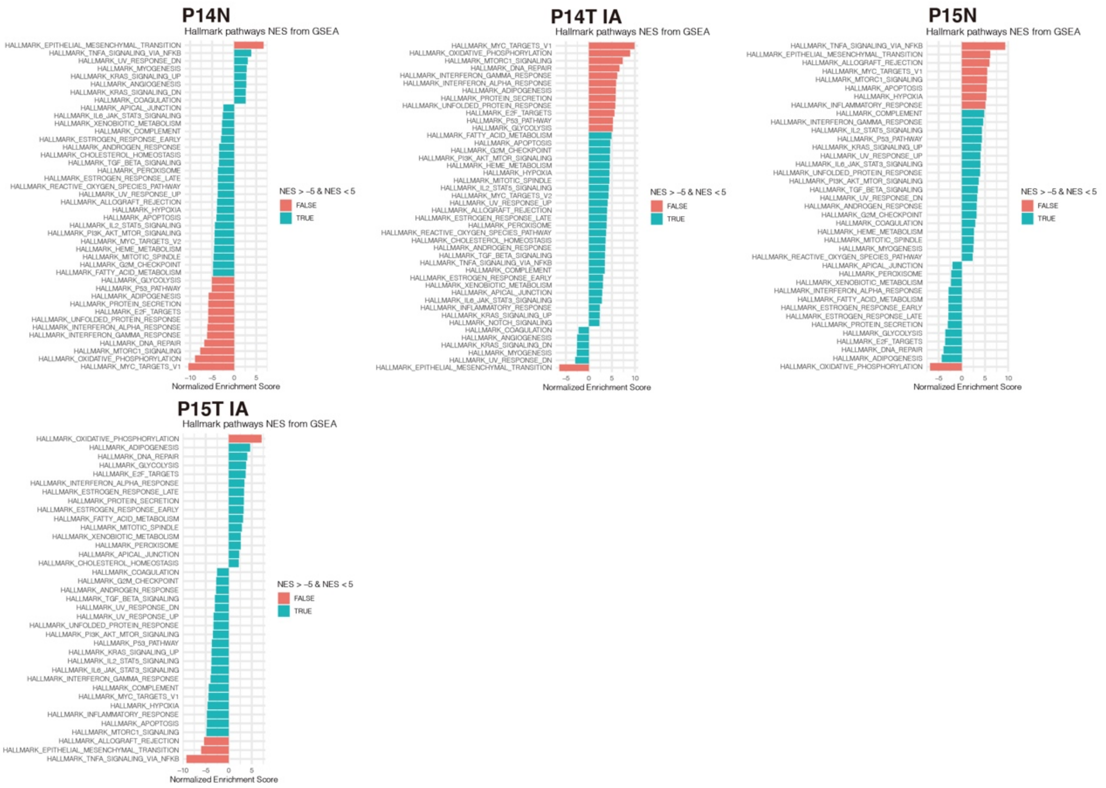

Supplementary Data File 9. Top enriched pathway of endothelial cells isolated from normal or tumor tissues of individual patients as determined by GSEA. The labels indicate tumor identifiers.
