# Supplementary material for: Deciphering cell lineage specification of human lung adenocarcinoma with single-cell RNA sequencing
Source: Nat Commun. 2021 Nov 11;12:6500. doi: 10.1038/s41467-021-26770-2 (PMC8586023; doi:10.1038/s41467-021-26770-2)
Supplement: Supplementary file 12 — Reporting Summary [file 41467_2021_26770_MOESM12_ESM.pdf]

nature research

Corresponding author(s): Weimin Li

Last updated by author(s): Jun 1, 2021

Reporting Summary

Nature Research wishes to improve the reproducibility of the work that we publish. This form provides structure for consistency and transparency in reporting. For further information on Nature Research policies, see our Editorial Policies and the Editorial Policy Checklist.

Statistics

For all statistical analyses, confirm that the following items are present in the figure legend, table legend, main text, or Methods section.

n/a	Confirmed	
☐	☒	The exact sample size (*n*) for each experimental group/condition, given as a discrete number and unit of measurement
☐	☒	A statement on whether measurements were taken from distinct samples or whether the same sample was measured repeatedly
☐	☒	The statistical test(s) used AND whether they are one- or two-sided<br>*Only common tests should be described solely by name; describe more complex techniques in the Methods section.*
☒	☐	A description of all covariates tested
☐	☒	A description of any assumptions or corrections, such as tests of normality and adjustment for multiple comparisons
☐	☒	A full description of the statistical parameters including central tendency (e.g. means) or other basic estimates (e.g. regression coefficient) AND variation (e.g. standard deviation) or associated estimates of uncertainty (e.g. confidence intervals)
☐	☒	For null hypothesis testing, the test statistic (e.g. *F*, *t*, *r*) with confidence intervals, effect sizes, degrees of freedom and *P* value noted<br>*Give P values as exact values whenever suitable.*
☒	☐	For Bayesian analysis, information on the choice of priors and Markov chain Monte Carlo settings
☒	☐	For hierarchical and complex designs, identification of the appropriate level for tests and full reporting of outcomes
☒	☐	Estimates of effect sizes (e.g. Cohen's *d*, Pearson's *r*), indicating how they were calculated

Our web collection on statistics for biologists contains articles on many of the points above.

Software and code

Policy information about availability of computer code

Data collection

The transcriptome data of TCGA LUAD were collected from the following web-links https://portal.gdc.cancer.gov/projects/TCGA-LUAD. The human-specific databases for RcisTarget were downloaded from (https://resources.aertslab.org/cistarget/databases/homo_sapiens/hg19/refseq_r45/mc9nr/gene_based/hg19-500bp-upstream-7species.mc9nr.feather) and (https://resources.aertslab.org/cistarget/databases/homo_sapiens/hg19/refseq_r45/mc9nr/gene_based/hg19-tsscentered-10kb-7species.mc9nr.feather).

Data analysis

BWA-mem (version 0.7.13-r1126),
SAMtools (version 1.9)
Picard Tools (version 2.2.1)
The Genome Analysis Toolkit (GATK, version 4.1.2.0)
ANNOVAR (version 2020.06.07)
CellRanger (version 3.0.0) were combined in R (version 3.6.3)
Seurat R package (version 3.0.3.9028) with the specified “Regress Out”, “FindClusters” function.
fgsea package (version 1.8.0)
msigdbr package (version 7.2.1)
Monocle2 (version 2.12.0)
inferCNV (version 1.0.4)
UPhyloplot2 (version 2.3)
bigSCale2 (version 2.0)
Cytoscape (version 3.8.0)
CellPhoneDB (version 2.1.2)
R package SCENIC (version 1.1.2)
Bioconductor TCGA biolinks package (version 2.2.10)
R packages “survival” (version 3.2.3) and “survminer” (version 0.4.7).
Phenoptics inForm software (version 2.4.8)

Caseviewer software (version 2.3)
SPSS software (version18.0)

For manuscripts utilizing custom algorithms or software that are central to the research but not yet described in published literature, software must be made available to editors and reviewers. We strongly encourage code deposition in a community repository (e.g. GitHub). See the Nature Research guidelines for submitting code & software for further information.

Data

Policy information about availability of data

All manuscripts must include a data availability statement. This statement should provide the following information, where applicable:

- Accession codes, unique identifiers, or web links for publicly available datasets
- A list of figures that have associated raw data
- A description of any restrictions on data availability

The generated WES, WGS and RNA-seq data in this study have been deposited to Genome Sequence Archive (GSA) in BIG Data Center, Beijing Institute of Genomics (BIG) under accession number HRA001130 [https://ngdc.cncb.ac.cn/gsa-human/browse/HRA001130]. The transcriptome data of TCGA LUAD were collected from the following web-links https://portal.gdc.cancer.gov/projects/TCGA-LUAD. The human-specific databases for RcisTarget were downloaded from (https://resources.aertslab.org/cistarget/databases/homo_sapiens/hg19/refseq_r45/mc9nr/gene_based/hg19-500bp-upstream-7species.mc9nr.feather) and (https://resources.aertslab.org/cistarget/databases/homo_sapiens/hg19/refseq_r45/mc9nr/gene_based/hg19-tsscentered-10kb-7species.mc9nr.feather). Source data are provided with this paper.

Field-specific reporting

Please select the one below that is the best fit for your research. If you are not sure, read the appropriate sections before making your selection.

☒ Life sciences ☐ Behavioural & social sciences ☐ Ecological, evolutionary & environmental sciences

For a reference copy of the document with all sections, see nature.com/documents/nr-reporting-summary-flat.pdf

Life sciences study design

All studies must disclose on these points even when the disclosure is negative.

Sample size	A total of 34 freshly resected lung tumor specimens were collected from 25 patients in different histologic subtypes of LUAD (3 AAH, 5 AIS, 9 MIA, and 17 IA) along with 18 adjacent normal lung tissues from a distal region within the same lobe, which served as controls. At least 3 samples were included in each group.
Data exclusions	Cells were removed if they had more than 20,000 UMIs, more than 3,000 or fewer than 300 expressed genes, or >10% UMIs that were derived from the mitochondrial genome.
Replication	Samples were processed for scRNA-seq and staining at least from three samples. We characterized the transcriptome of 140,556 cells from patients P1-P22 at single-cell resolution using the V2 kits and validated our results on a separate dataset of 127,923 single cells from multiple nodules in patients P23-P25 processed using the V3 kits. Biological replicates and technical replicates are all concordant.
Randomization	The patients were randomly included. According to the pathological diagnosis after operation, the tumors were divided into four groups (AAH, AIS, MIA and IA), and at least 3 samples were included in each group.
Blinding	Single-cell clustering and tumor cell lineage inference, as well as survival analysis, were all via unsupervised or blinded approaches.

Reporting for specific materials, systems and methods

We require information from authors about some types of materials, experimental systems and methods used in many studies. Here, indicate whether each material, system or method listed is relevant to your study. If you are not sure if a list item applies to your research, read the appropriate section before selecting a response.

Materials & experimental systems

n/a	Involved in the study	
☐	☒	Antibodies
☒	☐	Eukaryotic cell lines
☒	☐	Palaeontology and archaeology
☒	☐	Animals and other organisms
☐	☒	Human research participants
☒	☐	Clinical data
☒	☐	Dual use research of concern

Methods

n/a	Involved in the study	
☒	☐	ChIP-seq
☐	☒	Flow cytometry
☒	☐	MRI-based neuroimaging

Antibodies

Antibodies used	SFTPC (Millipore, AB3786, 1:200), SFTPA (Millipore, AB3420-I, 1:200), AGER (R&D, AF1145, 1:50), SCGB1A1 (Santa Cruz, SC-365992, 1:100), FOXJ1(Abcam, ab235445, 1:200), Nkx2-1(Millipore, SAB1403709, 1:500), TIMP1 (Invitrogen, MA5-13688, 1:200), EPCAM (Abcam, ab223582, 1:50). CD45 (Servicebio, GB14038, 1:100), CD31(Servicebio, GB14033, 1:200), VEGF (Servicebio, GB14165, 1:200), Vimentin (Servicebio, GB111308, 1:1500), Fibronectin (Servicebio, GB13091, 1:100), Ki67 (Servicebio, GB14102, 1:200), and MDK (Abcam, ab215835, 1:50). The secondary antibodies were Alexa Fluor 488-conjugated AffiniPure Donkey Anti-Rabbit IgG H+L min X Bovine, Chicken, Goat, Guinea Pig, Syrian Hamster, Horse, Human, Mouse, Rat, Sheep Serum Proteins Jackson, 711-545-15, 1:500 ; Cy3-conjugated AffiniPure Donkey Anti-Mouse IgG H+L min X Bovine, Chicken, Goat, Guinea Pig, Syrian Hamster, Horse, Human, Rabbit, Rat, Sheep Serum Proteins, Jackson, 715-165-15, 1:500); Alexa Fluor 647-conjugated AffiniPure Donkey Anti-Goat IgG H+L min X Chicken, Guinea Pig, Syrian Hamster, Horse, Human, Mouse, Rabbit, Rat Serum Proteins, Jackson, 705-605-14, 1:500). Antibodies in the Opal Polaris 7-Color Manual IHC were in working dilution, including Pan CK (clone AE1/AE3), FoxP3 (clone D608R), PD-L1 (clone E1L3N), PD-1 (clone, EPR4877), CD8 (clone 4B11), and CD68 (clone PG-M1)
Validation	Validated by the manufacturer and in comparison to negative/isotype controls. No validation statements for the antibodies that are commercially available.

Human research participants

Policy information about studies involving human research participants

Population characteristics	A total of 25 patients with different histologic subtypes of LUAD (3 AAH, 5 AIS, 9 MIA, and 17 IA) were included in this study. Pathology results were verified independently by two experienced LUAD pathologists. In addition, CT images of all patients were re-reviewed by an experienced imaging physician. Patients were diagnosed with AAH, AIS, MIA, or IA according to the 2015 WHO classification. These patients included 7 males and 18 females with an average age of 56 (range 38-78).
Recruitment	This is a retrospective study. We collected bio-specimens from Department of Thoracic Surgery, West China Hospital, China. The patients were randomly included. According to the pathological diagnosis after operation, the tumors were divided into four groups (AAH, AIS, MIA and IA).
Ethics oversight	This study was approved by the local ethics committee at West China Hospital of Sichuan University (Ethics: project identification code: 2018.270), and procedures complied with all relevant ethical regulations.

Note that full information on the approval of the study protocol must also be provided in the manuscript.

Flow Cytometry

Plots

Confirm that:

☒ The axis labels state the marker and fluorochrome used (e.g. CD4-FITC).

☒ The axis scales are clearly visible. Include numbers along axes only for bottom left plot of group (a 'group' is an analysis of identical markers).

☒ All plots are contour plots with outliers or pseudocolor plots.

☐ A numerical value for number of cells or percentage (with statistics) is provided.

Methodology

Sample preparation	Resected tumors were transported in Hank's Balanced Salt Solution (HBSS, Life Technologies) on ice immediately after surgery. The tumor sample was subsequently divided into two pieces, and a small fragment was stored in liquid nitrogen for tissue staining. The remainder of the tumor was minced with scalpels into tiny cubes <0.5 mm3 and transferred into a 15-mL conical tube (BD Falcon) containing 8 mL pre-warmed HBSS, 1 mg/mL collagenase I and 0.5 mg/mL collagenase IV. Tumor pieces were digested on a Tube Revolver (Thermo) for 30 min at 37 °C. This suspension was then filtered using a 70-µm nylon mesh (BD Biosciences) and residual cell clumps were discarded, then the cell pellet was resuspended in red blood cell lysis buffer. Following a 5-min incubation at room temperature, samples were centrifuged to discard the supernatant and re-suspend the cell pellet in PBS with 0.04% FBS. Cell sorting was performed with a MoFloAstrios EQ (Beckman Coulter). Live cells were used for single-cell experiments after the dead cells were eliminated based on exclusion of 7-aminoactinomycin D (Life Technologies).
Instrument	Cell sorting was performed with a MoFloAstrios EQ (Beckman Coulter). Live cells were used for single-cell experiments after the dead cells were eliminated based on exclusion of 7-aminoactinomycin D.
Software	Data was analyzed using FlowJo v10 software.
Cell population abundance	Cell sorting was performed to remove the dead cells, and 268,471 cells collected from 25 patients in four histologic stages of LUAD were used for analyzing.
Gating strategy	Cells sorting was performed to remove the dead cells based on exclusion of 7-aminoactinomycin D.

☐ Tick this box to confirm that a figure exemplifying the gating strategy is provided in the Supplementary Information.
